# Kinetics of Coinfection with Influenza A Virus and *Streptococcus pneumoniae*

**DOI:** 10.1371/journal.ppat.1003238

**Published:** 2013-03-21

**Authors:** Amber M. Smith, Frederick R. Adler, Ruy M. Ribeiro, Ryan N. Gutenkunst, Julie L. McAuley, Jonathan A. McCullers, Alan S. Perelson

**Affiliations:** 1 Department of Infectious Diseases, St. Jude Children's Research Hospital, Memphis, Tennessee, United States of America; 2 Departments of Mathematics and Biology, University of Utah, Salt Lake City, Utah, United States of America; 3 Theoretical Biology and Biophysics, Los Alamos National Laboratory, Los Alamos, New Mexico, United States of America; 4 Instituto de Medicina Molecular, Faculdade de Medicina da Universidade de Lisboa, Lisboa, Portugal; 5 Department of Molecular and Cellular Biology, University of Arizona, Tucson, Arizona, United States of America; 6 Department of Immunology and Microbiology, University of Melbourne, Victoria, Australia; Princeton University, United States of America

## Abstract

Secondary bacterial infections are a leading cause of illness and death during epidemic and pandemic influenza. Experimental studies suggest a lethal synergism between influenza and certain bacteria, particularly *Streptococcus pneumoniae*, but the precise processes involved are unclear. To address the mechanisms and determine the influences of pathogen dose and strain on disease, we infected groups of mice with either the H1N1 subtype influenza A virus A/Puerto Rico/8/34 (PR8) or a version expressing the 1918 PB1-F2 protein (PR8-PB1-F2(1918)), followed seven days later with one of two *S. pneumoniae* strains, type 2 D39 or type 3 A66.1. We determined that, following bacterial infection, viral titers initially rebound and then decline slowly. Bacterial titers rapidly rise to high levels and remain elevated. We used a kinetic model to explore the coupled interactions and study the dominant controlling mechanisms. We hypothesize that viral titers rebound in the presence of bacteria due to enhanced viral release from infected cells, and that bacterial titers increase due to alveolar macrophage impairment. Dynamics are affected by initial bacterial dose but not by the expression of the influenza 1918 PB1-F2 protein. Our model provides a framework to investigate pathogen interaction during coinfections and to uncover dynamical differences based on inoculum size and strain.

## Author Summary

Influenza virus infected individuals often become coinfected with a bacterial pathogen and, consequently, morbidity and mortality are significantly increased. A better understanding of how these pathogens interact with each other and the host is of key importance. Here, we use data from infected mice together with mathematical modeling and quantitative analyses to understand how each pathogen influences the other, and how the 1918 influenza PB1-F2 protein and the bacterial strain and dose contribute to coinfection kinetics. We find that influenza viral titers increase when *Streptococcus pneumoniae* is present and that the bacteria establish and grow rapidly when influenza is present. Our model and analyses suggest that the influenza infection reduces the bacterial clearance ability of alveolar macrophages and that the subsequent *S. pneumoniae* infection enhances viral release from infected cells. These results provide new insights into the mechanisms of influenza coinfection and the differences in pathogenesis of influenza and *S. pneumoniae* strains.

## Introduction

Influenza A virus (IAV) infection is often complicated by bacterial invasion, particularly with *Streptococcus pneumoniae* (pneumococcus). This can render a mild influenza infection severe or even fatal [1]. Increased severity and case fatality rates when secondary bacterial pneumonia occurs as a complication of influenza have been emphasized by studies of the 1918, 1957, 1968 and 2009 influenza pandemics [1]–[3]. The mechanisms driving virulent influenza co-infection are poorly understood, making it difficult to develop effective therapeutic strategies. This is particularly important since antibiotic use has had little impact on the mortality rates of influenza-associated bacterial pneumonia [4]. An understanding of the mechanisms involved in the interaction between influenza and bacterial pathogens is essential to finding treatment regimens that combat both the influenza infection and the secondary bacterial infection.

Although damage and immunological changes in the respiratory tract environment resulting from an influenza infection undoubtedly aid bacterial acquisition, progression to viral and/or bacterial pneumonia also depends on host immune status, sequence and timing of infections, inoculum size, and pathogen type and strain [5]. To control for these variables, animal models that study the underlying contributing factors of the synergistic interaction have been developed [5]–[7]. In mice, it was found that the bacterial inoculum size needed to induce severe secondary bacterial pneumonia was lower than that needed to elicit clinical symptoms in a primary infection [6]. In particular, a recent study of ours showed that establishing a pneumococcal infection in naive mice in the absence of influenza required an inoculum of 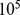 colony forming units (CFU) [8], while 7 days after influenza inoculation 100 CFU is sufficient to cause severe pneumonia [6].

Infection characteristics, including inflammation and airway destruction, are altered during coinfection with influenza and pneumococci [9], [10]. It has recently been shown that that an IAV infection can decrease mucociliary clearance of pneumococci *in vivo* [11]. In addition, pneumococci show increased adherence to lung epithelium in the presence of influenza, which could be mediated by viral neuraminidase (NA) activity [7], [10], [12]. Although improved adhesion is observed *in vitro* in cells damaged by toxic effects of influenza, the strength of this effect is reduced when less virulent viruses with lower NA activity are in circulation where there is still a high incidence of secondary infections [5].

The influence of host immune responses on these synergistic interactions has been studied more recently because the responses to influenza and pneumococcus use many of the same pathways, cofactors and intermediates [13]. Influenza has been shown to induce neutrophil apoptosis [14] and dysfunction [15], enhance bacterial-mediated apoptosis of phagocytic cells [16], and depress the macrophage-monocyte chemotactic and phagocytic functions [17]. Prior to and during coinfection, several proinflammatory cytokines, including 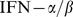 [18], [19], 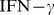, 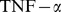, and IL-6 [20], [21], and the anti-inflammatory cytokine IL-10 [22], are significantly elevated and can influence downstream events such as macrophage and neutrophil recruitment [18], [19]. In general, IAV infection causes desensitization of immune responses [23], including systemic immune suppression [24].

Some evidence suggests that many of the aforementioned processes affect the later stages of secondary pneumococcal pneumonia rather than the initial clearance of bacterial populations [25]. To establish, pneumococci must first overcome resident alveolar macrophages, the initial line of cellular defense [26], [27], and then the neutrophils that appear several hours later [28]. Indeed, increased production of 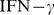 during the recovery from influenza reduces the ability of alveolar macrophages to phagocytose incoming bacteria [25], [29], [30], which then contributes to a dysfunctional neutrophil response [23]. The end result is an amplified response that is not effective in clearing bacterial populations and increases pathogenesis, despite large numbers of neutrophils and macrophages in the lung [5].

The IAV protein PB1-F2 [31] has been linked to these effects on neutrophils and macrophages and may increase the pathologic tissue destruction observed during a bacterial superinfection [9], [32]. We and others have found that PB1-F2 induces large infiltrates of immune cells [9], [33], [34] and significantly increases the establishment of secondary bacterial pneumonia *in vivo* [9]. Furthermore, PB1-F2 can modulate the type I interferon response in infected cells [33], [35], resulting in increased infiltration of monocytes and neutrophils [33]. However, PB1-F2 expression is also connected to the apoptosis of IAV-infected monocytes [31], [36], [37]. This may help to explain the pathogenicity of the 1918 influenza pandemic since a virus engineered to express the 1918 PB1-F2 protein was more virulent and induced a strong inflammatory response during secondary pneumococcal pneumonia [9].

Together, these findings emphasize that several factors contribute to the enhanced susceptibility of influenza infected individuals to secondary bacterial infections. However, the primary focus of research thus far has been on understanding how influenza affects the subsequent bacterial infection. Viral loads in the lungs following bacterial challenge and the mechanisms responsible for any changes have not been carefully studied [6]. Determining the extent to which resolution of the influenza infection is altered is critical to fully understanding the synergistic relationship between influenza and its bacterial counterparts.

To examine possible mechanisms and provide links to their relative effects, mathematical models can be used to tease apart the effects of each mechanism on viral and bacterial lung titers. Several studies have used kinetic models to study influenza virus kinetics and the associated immune responses (reviewed in [38]–[40]) but only one study has mathematically modeled pneumococcal dynamics [8]. These models have yet to be combined to assess coinfection dynamics.

This study presents both empircal data on coinfection dynamics and modeling of such a combined model. We first examine lung titer data collected from groups of BALB/cJ mice that were infected with 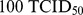 influenza A virus A/Puerto Rico/8/34 (H1N1) or a variant expressing the 1918 PB1-F2 protein (PR8-PB1-F2(1918)), and then 7 days later infected with 100 CFU or 1000 CFU *S. pneumoniae* strain D39 (type 2) or with 1000 CFU *S. pneumoniae* strain A66.1 (type 3). These data show an important consequence of pneumococcal coinfection with influenza, increased viral titers. Our kinetic model describing coinfection can evaluate hypothesized mechanisms of interaction and study the effects of (i) the bacterial inoculum size (100 CFU versus 1000 CFU D39) and (ii) the virus strain (PR8 versus PR8-PB1-F2(1918)) on acquisition and infection kinetics of a secondary bacterial infection.

## Results

### Experimental results

**Figure 1:**
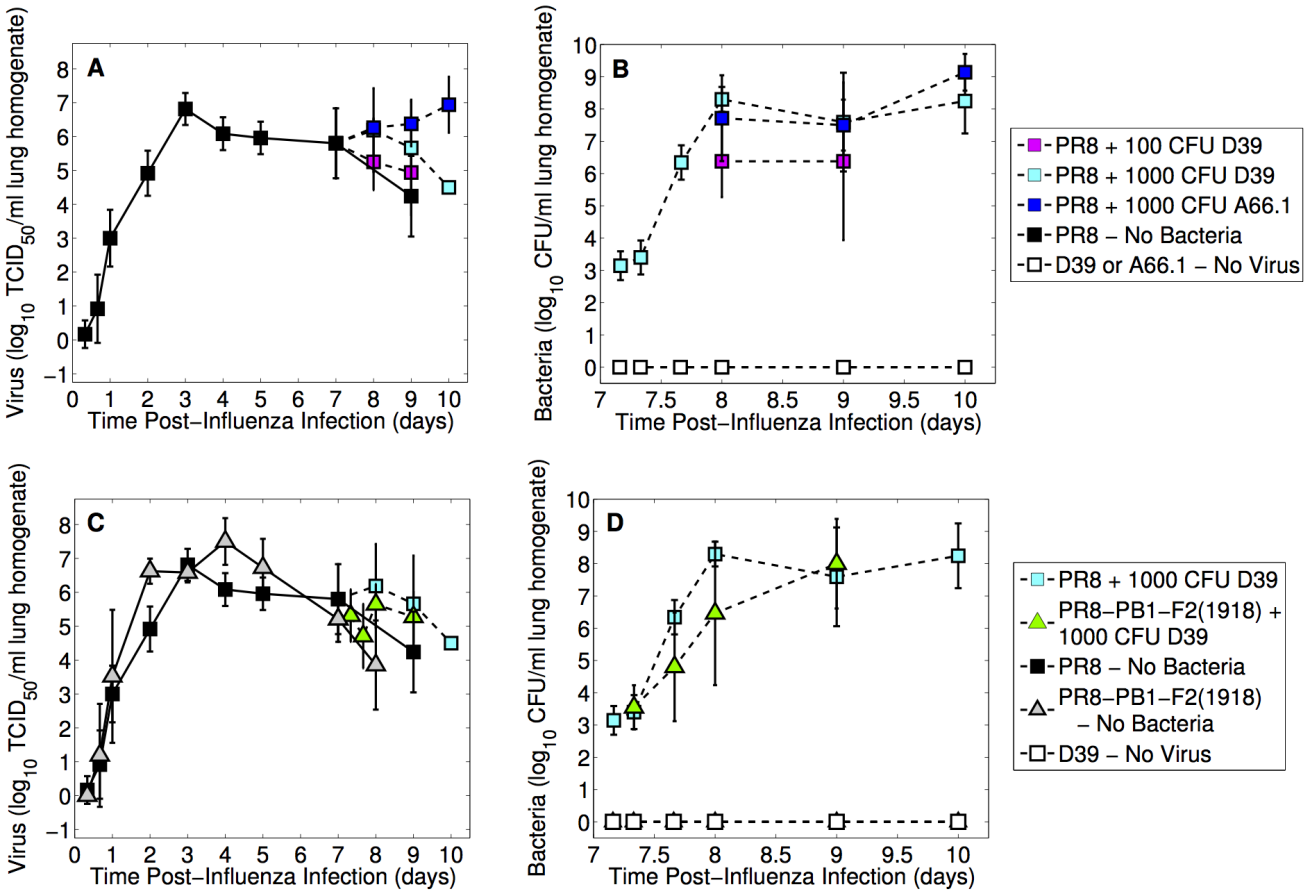
Lung viral and bacterial titers from mice infected with influenza A virus then infected 7 days later with pneumococcus. In the first set of experiments (Panels A and B), mice were infected with 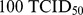 of influenza A virus PR8 then 7 days later with either *S. pneumoniae* strain D39 (100 CFU or 1000 CFU) or A66.1 (1000 CFU). Panels C and D show comparable experiments in which mice were infected with 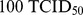 of influenza A virus PR8-PB1-F2(1918) then 7 days later with D39 (1000 CFU). Control experiments in which mice were infected with only virus or only bacteria are also shown. Data are given as geometric means 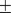 SD.

Influenza lung titers (Figure 1A,C), for both PR8 and PR8-PB1-F2(1918), initially increase exponentially reaching maximum titers of 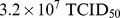/ml lung homogenate and 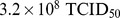/ml lung homogenate, respectively [41]. Mice inoculated with PR8 had viral titers peaking at 72 hours post inoculation (p.i.) while mice given the PR8-PB1-F2(1918) virus reached high titers (equivalent to the peak of PR8) slightly earlier at 48 hours p.i. (Figure 1C). However, PR8-PB1-F2(1918) values remain high through 4 days p.i.. Viral titers of both strains then decline as the mice begin to recover.

#### Viral titer rebound

On day 7 when the bacterial challenge is given, viral titers for PR8 and PR8-PB1-F2(1918) are one and two logs lower, respectively, on average than their peaks. Although the viral titers of PR8 and PR8-PB1-F2(1918) remain statistically indistinguishable following bacterial challenge (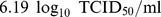 versus 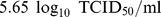 at 8 days p.i., p = 0.30, and 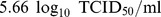 versus 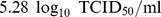 at 9 days p.i, p = 0.50), the dynamics of each virus strain is altered by bacterial presence. Rather than decaying, a second viral titer peak is evident for both strains at a bacterial inoculum of 1000 CFU of pneumococcal strain D39 (Figure 1C), where viral titers increase by a factor of 3 following PR8 infection and by a factor of 2.5 following PR8-PB1-F2(1918) infection.

In the absence of bacteria, the PR8 viral titer 9 days p.i. is 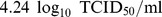, whereas the viral titer following inoculation with 1000 CFU D39 is 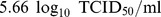 (p = 0.024) and following 1000 CFU A66.1 is 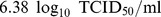 (p = 0.0014). Similarly, for influenza infection with PR8-PB1-F2(1918), the difference at 8 days p.i. with a bacterial challenge of 1000 CFU D39 on day 7 is significant compared to the bacteria-free infection (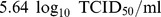 versus 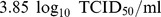, p = 0.019). The rebound of influenza titers is less evident and not statistically significant for inoculation with 100 CFU D39 following PR8 (Figure 1A) compared to no bacterial inoculation (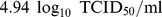 versus 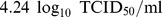 at 9 days p.i., p = 0.38).

#### Sustained bacterial titer

Pneumococcal lung titers following PR8 viral infection rise quickly within 24 hours (Figure 1B) and reach maximum values of 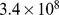 CFU/ml for challenge with 100 CFU D39, 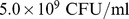 for challenge with 1000 CFU D39, and 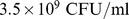 for challenge with 1000 CFU A66.1. These are significantly elevated compared to mice infected with either 100 CFU or 1000 CFU in the absence of a viral infection (Figure 1B,D), where the bacterial titers for either strain were already undetectable 4 hours p.i. for bacterial inocula less than 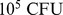 [8], [21], [42].

A larger bacterial inoculum (1000 CFU versus 100 CFU) of pneumococcal strain D39 following PR8 infection resulted in significantly higher bacterial titers 8 days p.i. (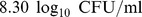 versus 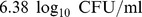, p = 0.003) that remained high throughout the course of infection although the difference between these inocula 9 days p.i. was not significant (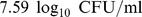 versus 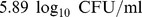, p = 0.16). There were no differences in bacterial titers of mice inoculated with D39 (1000 CFU) and those with A66.1 (1000 CFU), where  titers (CFU/ml) at 8, 9 and 10 days p.i. were 8.30 and 7.72 (p = 0.18), 7.59 and 7.50 (p = 0.85), and 8.25 and 9.14 (p = 0.11), respectively (Figure 1B). Bacterial titers in mice infected with PR8-PB1-F2(1918) reached maximum values of 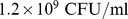 but were not statistically different from mice infected with PR8 (Figure 1D), where 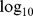 titers (CFU/ml) at 8, 16, 24, and 48 hours post-bacterial infection were 3.55 and 3.40 (p = 0.68), 4.80 and 6.34 (p = 0.08), 6.46 and 8.30 (p = 0.10), and 8.0 vs 7.59 (p = 0.60).

**Figure 2:**
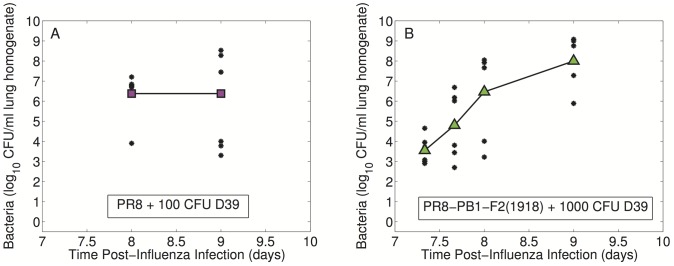
Dichotomy of bacterial lung titers. Individual (black dots) and average (colored boxes/triangles) 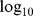 values of bacterial titers to illustrate the generation of high or low titers that can occur in some infections. Mice were infected with either (A) 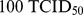 PR8 virus followed 7 days later by 100 CFU *S. pneumoniae* strain D39 or (B) 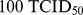 PR8-PB1-F2(1918) virus followed 7 days later by 1000 CFU *S. pneumoniae* strain D39.

In two of the data sets, mice broke into two categories: one group that developed high bacterial titers and one that developed significantly lower bacterial titers (Figure 2). This phenomenon occurred predominantly for PR8 infection followed by 100 CFU D39 at 9 days p.i. and for PR8-PB1-F2(1918) infection followed by 1000 CFU D39 at 8 days p.i.. Recovery, as measured by weight gain, was not observed in any of our experiments.

### Coinfection model results

**Figure 3:**
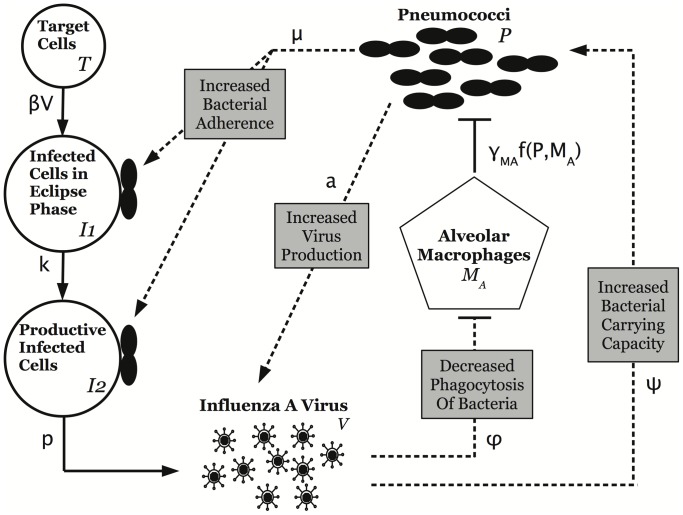
Schematic diagram of the coinfection model dynamics. Dashed lines indicate the interactions between influenza and pneumococcus, including (i) increased bacterial adherence to infected cells, (ii) increased infected cell death from bacterial adherence, (iii) viral-induced decrease in phagocytosis of bacteria, and (iv) bacterial-induced increase in virus release.

To investigate the underlying mechanisms of lethal synergy between influenza and pneumococcus, we developed a kinetic model (Equations (6)–(10)) based on proposed mechanisms of interaction between these two pathogens. We consider two viral effects that may enhance the secondary bacterial infection, i.e., increased bacterial adherence to epithelial cells and alveolar macrophage dysfunction, and two bacterial effects that may influence the viral coinfection, i.e., increased viral release from infected epithelial cells and increased infected cell death from bacterial adherence. The model schematic is in Figure 3.

**Figure 4:**
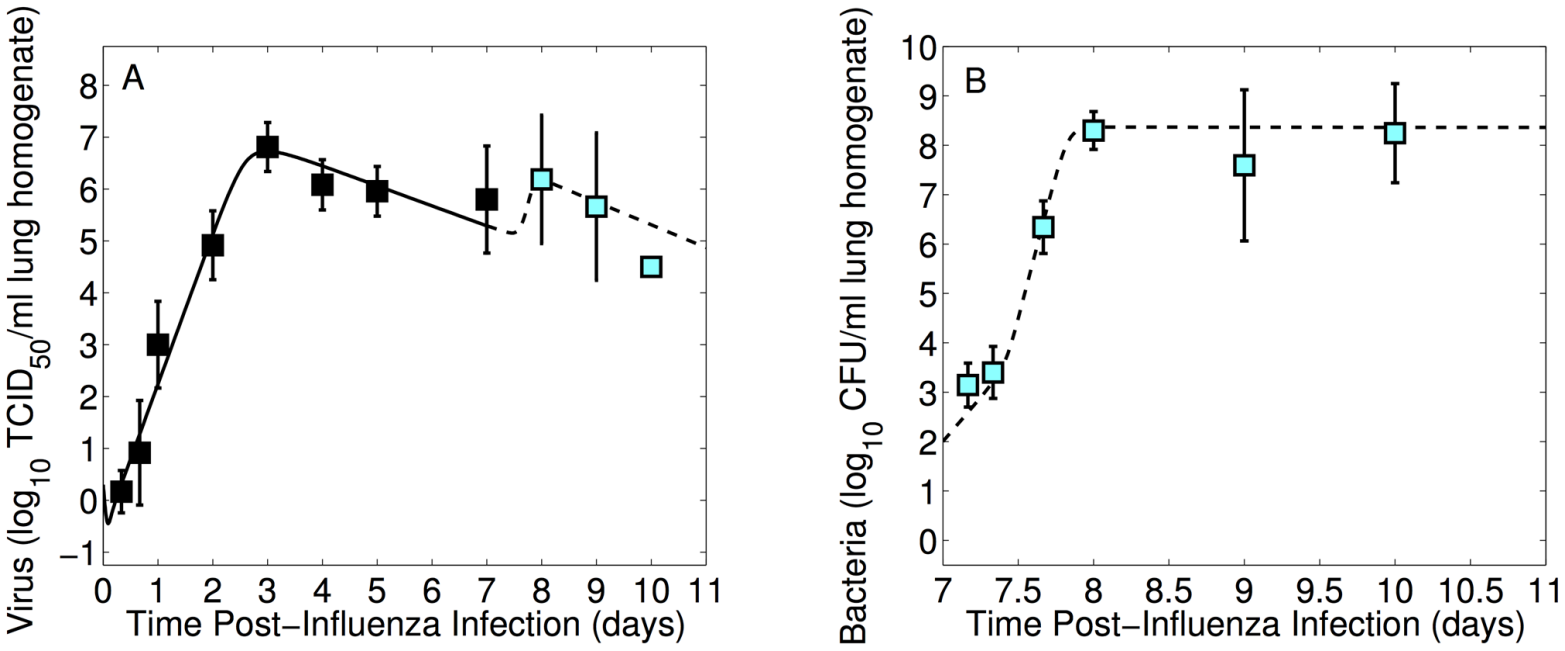
Coinfection model fit to lung titers of mice coinfected with PR8 and 1000 CFU D39. Fit of the coinfection model (Equations (6)–(10)) to viral (panel A) and bacterial (panel B) lung titers from individual mice infected with 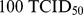 PR8 virus followed 7 days later by 1000 CFU *S. pneumoniae* strain D39. Parameters for the model curves are in Tables 1–2.

We use the lung titers obtained from mice infected 7 days after PR8 inoculation with 1000 CFU D39 to fit the coinfection model, Equations (6)–(10), simultaneously to the lung viral and bacterial titers. To do this, we fixed the parameters corresponding to models of infection with only influenza virus [41] or only pneumococcus [8] (Table 1) and set the initial value for an inoculum of 1000 CFU to 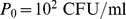 (see Materials and Methods). We then estimated the coinfection parameters, which are shown in Table 2, together with the associated 95% confidence intervals. The model fit is in Figure 4. To study the influence of parameters on our results, we also performed a Bayesian ensemble analysis [43] and a sensitivity analysis [44], [45] (details in Text S1). Taken together, several important aspects of the dynamics are highlighted.

**Table 1:** Previously established parameter values of the influenza model (Equations (1)–(4)) [41] and the pneumococcus model (Equation (5)) [8].

Parameter	Description	Units	Value	
Influenza A Virus			**PR8**	**PR8-PB1-F2(1918)**
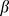	Virus infectivity	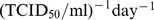	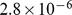	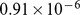
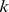	Eclipse phase	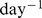	4.0	4.0
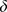	Infected cell death	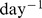	0.89	1.5
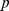	Virus production	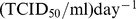	25.1	72.8
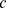	Virus clearance	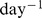	28.4	9.2
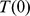	Initial uninfected cells	cells	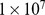	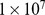
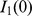	Initial infected cells	cells	0	0
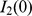	Initial infected cells	cells	0	0
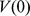	Initial virus	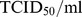	2.0	0.26
Pneumococcus			**D39**	
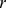	Bacterial growth rate	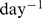	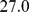	
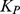	Carrying capacity	CFU/ml	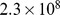	
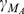	Phagocytosis rate	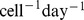	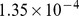	
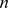	Maximum bacteria per AM	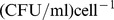	5.0	
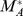	AM steady-state	cells	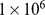	

**Table 2:** Parameter estimates and 95% confidence intervals from the coinfection model (Equations (6)–(10)) for the dynamics of infection with 1000 CFU D39 7 days after PR8 or PR8-PB1-F2(1918) infection.

Parameter	Description (Units)	Value	
**Viral Effects on Bacteria**			
		**PR8**	**PR8-PB1-F2(1918)**
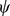	Increase in carrying capacity ( 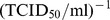 )	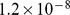	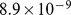
		[0, 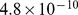 ]	[0, 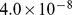 ]
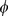	Decrease in phagocytosis rate	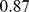	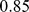
		[0.86, 0.91]	[0.85, 0.95]
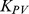	Half-saturation constant ( 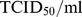 )	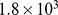	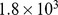
		[ 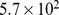 , 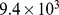 ]	[ 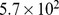 , 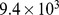 ]
**Bacterial Effects on Virus**			
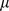	Toxic death of infected cells ( 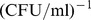 )	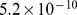	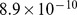
		[0, 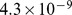 ]	[0, 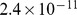 ]
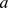	Increase in virion production/release ( 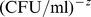 )	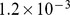	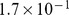
		[ 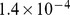 , 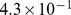 ]	[ 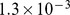 , 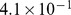 ]
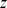	Nonlinearity of virion production/release	0.50	0.30
		[0.14, 0.61]	[0, 0.57]

* Because of the dependency between some parameters, there are many sets of parameters that give rise to equivalent fits (see Text S1).

Two of the four mechanisms we studied in our model significantly affected infection dynamics and two had only minor effects (Figures S5, S6, S7, S8, S9, S10, S11, S12, S13, S14, S15, S16, S17, S18, S19 in Text S1). First, the viral titer rebound observed shortly after the introduction of pneumococci, as illustrated in Figure 4A, could be explained as being a result of enhanced virion release from infected cells (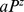) (Figures S6, S7, S12, S13 in Text S1). In our model, the viral decay that follows is a consequence of infected cells being lost to infection [46]. However, enhanced infected cell death from bacterial adherence (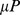) plays a minimal role and equivalent fits occur when this parameter is equal to 0 (Figures S1, S2, S3, S4, S5, S11 in Text S1).

As described, we also observed an enhanced growth of bacteria in the context of influenza coinfection. In the model, we found that a decreased rate of bacterial phagocytosis by alveolar macrophages in the presence of virus (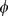) was sufficient to initiate rapid bacterial growth at a low inoculum (Figures S9, S10, S15, S16 in Text S1), suggesting that the protective effect of alveolar macrophages may be removed by the influenza infection. Furthermore, bacterial phagocytosis is initially taking place but quickly diminishes (within 10 hours post-bacterial challenge), as evidenced by the increase in slope of the bacterial curve (Figure 4B). Even as viral titers decline, bacterial titers remain significantly elevated and uncontrolled by alveolar macrophages. In the later stages of infection (i.e., 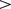
24 hours post-bacterial challenge), viral titers are decreasing and bacterial titers reach the maximum bacterial titer, 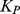 (Figure 4B). Interestingly, the viral-induced increase in bacterial carrying capacity (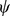) has minimal effects (Figures S1, S2, S3, S4, S8, S14 in Text S1) and can be set to 0 without significant impact on model dynamics (see Text S1).

### Infection with 100 CFU D39

**Figure 5:**
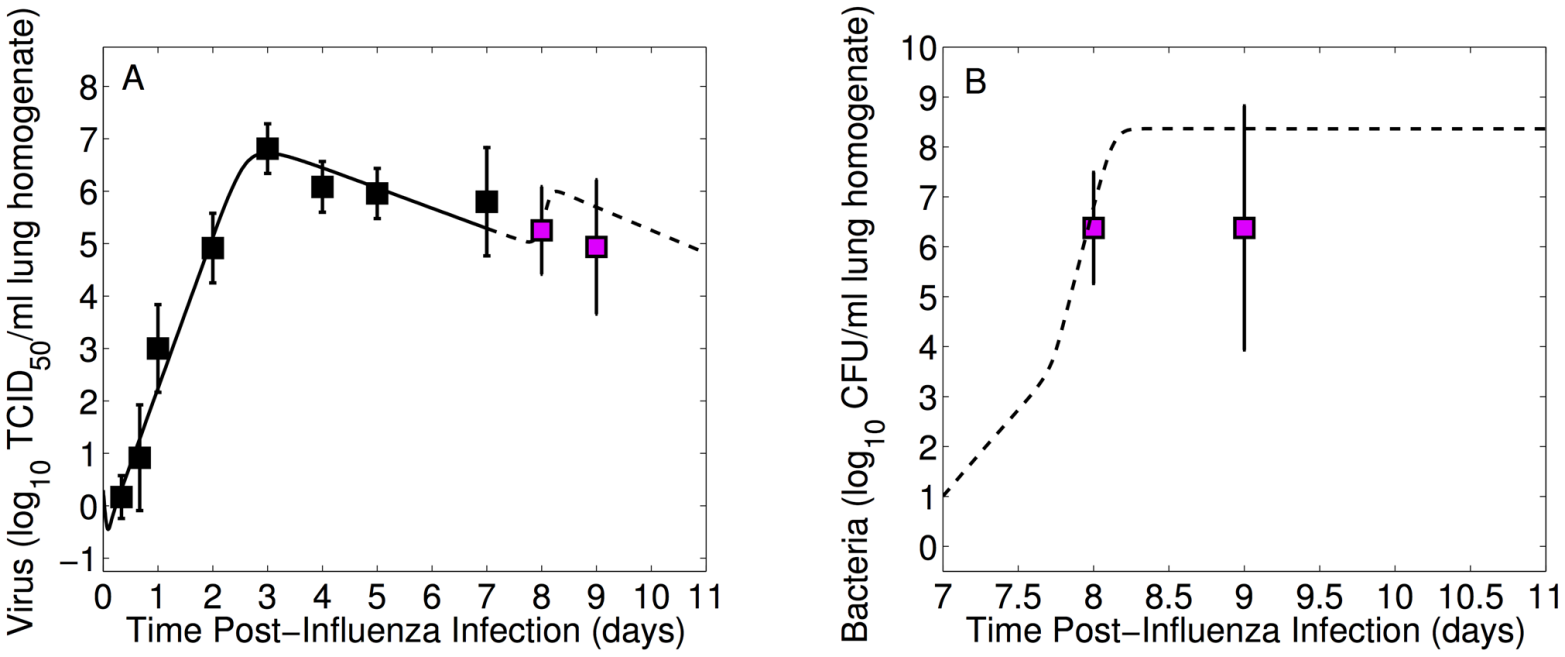
Simulation of the coinfection model with lung titers of mice coinfected with PR8 and 100 CFU D39. Numerical simulation of the coinfection model, Equations (6)–(10) with parameter values in Tables 1–2 against lung titers from individual mice infected with 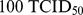 PR8 virus (panel A) followed 7 days later by 100 CFU *S. pneumoniae* strain D39 (panel B).

Using the parameters in Table 2, we simulated Equations (6)–(10) using a bacterial initial condition 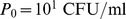, which corresponds to an inoculum of 100 CFU (see Materials and Methods) to evaluate the influence of bacterial inoculum size on infection kinetics. With a lower initial inoculum, the viral titer rebound is delayed (Figure 5A). Similarly, bacterial titers grow more slowly initially but then increase rapidly 17 hours post-bacterial challenge (Figure 5B), which corresponds with the increase in viral titers. In our model, bacterial titers reach a maximum carrying capacity (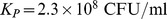) by 32 hours post-bacterial challenge. Average bacterial lung titers are much lower (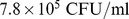) at this time point, but there are two distinct outcomes that are evident among the individual titer values, i.e., high or low bacterial titers. Our model predicts an outcome in which mice develop high bacterial titers. However, small perturbations in the amount of alveolar macrophage impairment (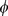) are sufficient to predict lower bacterial titers (Figures S9, S15 in Text S1).

### Expression of the 1918 influenza protein PB1-F2

**Figure 6:**
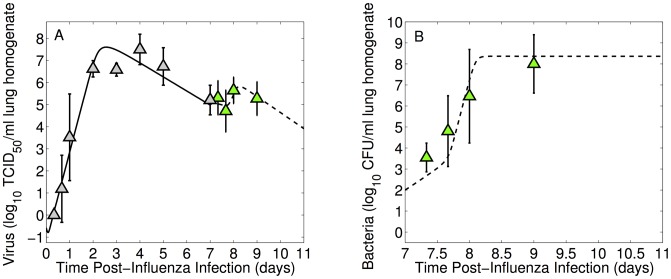
Coinfection model fit to lung titers of mice coinfected with PR8-PB1-F2(1918) and 1000 CFU D39. Fit of the coinfection model (Equations (6)–(10)) to viral (panel A) and bacterial (panel B) lung titers from individual mice infected with 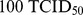 PR8-PB1-F2(1918) virus followed 7 days later by 1000 CFU *S. pneumoniae* strain D39. Parameters for the model curves are in Tables 1–2.

We then fit the coinfection model (Equations (6)–(10)) to the lung titers obtained from mice infected 7 days after PR8-PB1-F2(1918) inoculation with 1000 CFU D39. The model fit is in Figure 6 for the estimated parameters in Table 2. The estimated parameters varied only slightly from those estimated with PR8 infection, and indeed simulation of the coinfection model with the PR8 coinfection parameters (Table 2) and the viral parameters corresponding to an infection with PR8-PB1-F2(1918) (Table 1) also provides a good description of the experimental observations without further parameter adjustment (not shown).

Expression of the 1918 PB1-F2 protein leads to only slightly different infection kinetics during the coinfection phase. These effects primarily result from the differences during viral kinetics prior to bacterial challenge. Although the difference in viral lung titers between PR8 and PR8-PB1-F2(1918) 7 days p.i. was not statistically significant, the lower initial starting value of the PR8-PB1-F2(1918) virus at this time point may drive a lag in viral increase. Our model predicts that the second peak in PR8-PB1-F2(1918) viral titers is slightly lower and occurs 30 hours post-bacterial challenge as compared to 23 hours during infection with PR8. The bacterial titers also experience slower growth compared to PR8 coinfection, where bacterial phagocytosis occurs for an additional 6 hours with PR8-PB1-F2(1918) before bacterial titers reach the maximum tissue carrying capacity (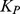) as viral titers decline.

## Discussion

Morbidity and mortality associated with pneumonia occurring from a bacterial infection associated with influenza remain high despite the availability and use of effective antivirals against influenza and antibiotics against *S. pneumoniae*. Even with development of animal models that facilitate the investigation of mechanisms which underlie pathogen interactions [5]–[7], [47]–[51], the numerous factors involved make examining each one in detail difficult. Establishing the roles of the interrelated contributions of the influenza infection, the pneumococcal invasion, and the host immune response without testing every scenario could aid in developing appropriate hypotheses for experimental testing and potentially improved treatment regimens.

We highlight two important features of the influenza-pneumococal coinfection, namely a rebound of viral titers post-bacterial inoculation and generation of either high or low bacterial titers possibly resulting in distinct outcomes. One mechanism suggested for the observed increase of viral titers after bacterial infection is the promotion of influenza virus fusion and entry into host cells via bacterial proteases [6], although detailed studies of the mechanism(s) driving this phenomena have not been performed. We examined this hypothesis by including an increase of viral infectivity (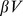) in the presence of pneumococci, however the number of susceptible target cells (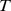) is low 7 days after influenza inoculation and thus including this effect in our model did not influence viral dynamics. The reduction in viral clearance is observed within hours after bacterial inoculation and the viral rebound peaks within 24 hours, suggesting a fast acting mechanism. The observed rebound of viral titers could be due to either a severe decrease in viral clearance (likely T-cell mediated clearance of infected cells) or a sudden burst of viral release. We explored both hypotheses within our kinetic model and found that increased virus production/release when pneumococci interact with influenza-infected epithelial cells could better explain the observed behavior. However, more work is necessary to pinpoint the driving mechanism, with bacterial proteases or bacterial NA likely factors.

Interestingly, coinfection with the serotype 3 pneumococcus A66.1 resulted in distinct viral titer dynamics from that of the type 2 pneumococcus D39, despite having comparable bacterial titers. Mice coinfected with A66.1 exhibited viral titers that continually increased over the infection time course. These two strains differ in that A66.1 is restricted to the lungs while D39 can become systemic. Thus, growth and/or clearance rates may differ between these strains. With more than 90 pneumococcal serotypes, the virulence and the host immune response vary between serotypes [52]. Comparing the dynamics of several pneumococcal strains, particularly with a kinetic model, both in the presence and absence of a viral infection would give important insight into the pathogenesis and variety of outcomes observed in different viral-bacterial pairings [53], [54]. Doing so may also help elucidate why, under certain circumstances and with some influenza and bacterial strains, the bacterial infection can reduce rather than enhance viral titers during coinfection [55]–[57].

While an improved viral production/release can explain the viral titer behavior, our model identifies a viral-dependent dysfunction in alveolar macrophage phagocytosis of bacteria as the other dominant mechanism controlling the synergy between influenza and pneumococcus. Decreased phagocytosis during pneumococcal colonization is sufficient to allow establishment and immediate growth of bacteria. As the pneumococcal population reaches high titers, the effect AMs have becomes irrelevant and the bacterial lung titers reach a maximum tissue capacity (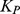). The close fit of our model to bacterial lung titer data for mice inoculated with 1000 CFU pneumococci (Figures 4–6) suggests that an additional influx of phagocytic cells would have negligible effects on bacterial removal. This may not be the case, however, with a lower bacterial inoculum (i.e., 100 CFU), where some mice had low titers 9 days p.i.. Thus, phagocytosis by neutrophils and/or recruited macrophages may be responsible for controlling lower doses of bacteria. We found similar dose dependent results for a pneumococcal infection in the absence of an antecedent viral infection [8]. In that model, which considered only the alveolar macrophage response, lung titers could be predicted up to 12 hours p.i., but a model including the neutrophil influx showed that this response was necessary to eliminate an inoculum of 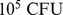 or higher of pneumococci.

In two of our data sets (PR8 +100 CFU D39 and PR8-PB1-F2(1918) +1000 CFU D39), we found that bacterial titers of individual mice followed one of two distinct patterns. The bacterial infection resulted in either very high bacterial titers or in very low bacterial titers. High bacterial titers are indicative of severe pneumonia, while low bacterial titers could suggest a mild infection and possibly even recovery. Although this could be due to experimental error, similar heterogeneity is observed in humans where some coinfections result in severe pneumonia while others are do not [58], [59]. Our model describes the severe infection but suggests that subtle differences in bacterial induced impairment of alveolar macrophage (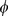) could explain this behavior (Figures S9, S13 in Text S1). Thus, decreasing this parameter can produce the dynamics of a mild infection in which lower bacterial titers are predicted. It is possible that this split is due to an early alveolar macrophage clearance phenotype, such that the infection is controlled overall if the inoculum is controlled in the first few hours via alveolar macrophage-mediated clearance, but uncontrolled exponential growth occurs thereafter if this threshold is exceeded. However, it is still interesting that this effect is dichotomous rather than continuous at the population level.

Population dynamics of alveolar macrophages during the primary influenza infection were excluded in our model because only a small percentage (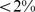) of alveolar macrophages infected with influenza undergo apoptosis [60]. However, 7 days after influenza inoculation, the infected lung will generally include several other cell types, e.g., neutrophils, recruited macrophages, and T-cells [61]. Although we have an accurate model of pneumococcal dynamics which includes neutrophils, recruited macrophages, proinflammatory cytokines, and tissue damage [8], we would need to develop a more complicated model of influenza dynamics in order to explore the effects of other cells and/or cytokines. To do so, significantly more data on each of these factors would be necessary so that the model could be validated.

Such a model would aid comparison between preinfection with PR8 and PR8-PB1-F2(1918) and provide information on how the cellular influx is altered by this protein. The differences we found between preinfection with PR8 and PR8-PB1-F2(1918) could be explained predominantly by the differences in viral parameters [41] rather than any changes that may come about during the bacterial coinfection. Although the inflammatory response has been shown to be enhanced during the secondary infection with expression of the 1918 PB1-F2 protein [9], our model cannot tease apart these effects on the host.

In modeling the coinfection kinetics, we are able to simultaneously evaluate whether several possible mechanisms can explain empirical observations by combining them into a single effect. This approach is convenient but cannot establish the exact mechanisms responsible. It does, however, aid experimental design by narrowing the focus to a particular biological process, as suggested in Table 3. For example, kinetic studies of alveolar macrophage phagocytic ability at various times during both influenza infection and secondary pneumococcal infection would expose how these cells influence bacterial acquisition. Our model suggests that distinct outcomes are possible such that with decreased alveolar macrophage inhibition (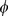), the bacterial infection would not establish. It is possible that as the influenza infection proceeds, the detrimental effects on alveolar macrophages accumulate over time and create various phenotypes. This may help to explain why the synergism between influenza and pneumococcus is maximal when influenza precedes pneumococcus and when inoculation with bacteria occurs 7 days after influenza [6].

**Table 3:** Summary of the coinfection model hypotheses, results and possible experiments to confirm each hypothesis.

Effect	Consequences	Hypothesis	Possible Experiments
**Alveolar Macrophage Dysfunction**	Decreased phagocytic ability, heterogeneity in individual lung titers, and loss of phagocytic cells and early innate immune signaling	Influenza-induces phenotypic changes and/or apoptosis in alveolar macrophages	Kinetic study of phagocyte numbers, recruitment, and differentiation states in the lungs and airways during influenza infection
**Enhanced Viral Release from Infected Cells**	Rebound of viral titers and altered immune responses	Bacterial proteases and/or neuraminidases affect viral release from infected cells	*In vitro* assay of virus production in the presence/absence of bacteria, and *in vivo* infections with viral-bacterial pairings that exhibit differential NA activity

Determining the upstream and downstream events related to the alveolar macrophage dysfunction and the subsequent neutrophil dysfunction is critical. These effects may, in part, be due to alterations in dendritic cells (DCs) during coinfection that result in an upregulation of proinflammatory cytokines (i.e., 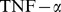, IL-12 and 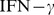) dependent on the time and dose of pneumococci [62]. However, elevated type I IFNs can inhibit the secretion of neutrophil chemoattractants KC and MIP-2 [19] and the macrophage chemoattractant CCL2 [18], which then influences the later stages of pneumococcal clearance. Other cells and co-factors may also play a role and have been implicated in modulating influenza virus coinfection with other bacteria (e.g., *Staphylococcus aureus*, *Listeria monocytogenes*, and *Bordetella pertussis*). These include (i) natural killer (NK) cells, which have an impaired response due to reduced 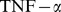 expression during coinfection with *S. aureus* [63], (ii) Th-17 cytokines IL-17, IL-22 and IL-23, which are significantly decreased possibly due to elevated type I IFNs (*S. aureus*) [64], (iii) elevated glucocorticoid levels, which lead to a sustained immunosuppression (*L. monocytogenes*) [24], and (iv) toxin-mediated disruption of the immune response to the virus (*B. pertussis*) [65]. More experiments and modeling studies are clearly necessary to further elucidate the factors driving the dynamics associated with influenza coinfection.

We have shown how coinfection with influenza and pneumococcus affects viral and bacterial titers and how these are influenced by changes in inoculum size and pathogen strain. We developed a kinetic model that predicted the behavior of lung titers and exposed two dominant factors influencing the interaction of these two pathogens. Although the synergy between influenza and pneumococcus involves many factors, identifying the most important processes in the protection against and the increased susceptibility to secondary infections may have a significant impact on the development of effective therapies.

## Materials and Methods

### Ethics statement

All experimental procedures were approved by the Animal Care and Use Committee at SJCRH under relevant institutional and American Veterinary Medical Association guidelines and were performed in a Biosafety level 2 facility that is accredited by AALAAS.

### Mice

Adult (6–8 wk old) female BALB/cJ mice were obtained from Jackson Laboratories (Bar Harbor, ME). Mice were housed in groups of 4–6 mice in high-temperature 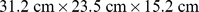 polycarbonate cages with isolator lids. Rooms used for housing mice were maintained on a 12∶12-hour light:dark cycle at 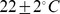 with a humidity of 50% in the biosafety level 2 facility at St. Jude Children's Research Hospital (Memphis, TN). Prior to inclusion in the experiments, mice were allowed at least 7 days to acclimate to the animal facility. Laboratory Autoclavable Rodent Diet (PMI Nutrition International, St. Louis, MO) and autoclaved water were available ad libitum. All experiments were performed under an approved protocol and in accordance with the guidelines set forth by the Animal Care and Use Committee at St. Jude Childrens Research Hospital.

### Infectious agents

Viruses used in the experimental model consist of (i) a mouse adapted Influenza A/Puerto Rico/8/34 (H1N1) (PR8), and (ii) a genetically engineered influenza virus referred to as “PR8-PB1-F2(1918).” The latter virus has a PR8 backbone with eight amino acid changes in the PB1 gene segment such that the virus expresses the PB1-F2 protein from influenza A/Brevig Mission/1/1918 (H1N1) as previously described [9], but is otherwise isogenic to PR8. *S. pneumoniae* strains D39 (type 2) and A66.1 (type 3) were transformed with the lux operon (Xenogen) to make them bioluminescent [10].

### Infection experiments

The viral dose infectious for 50% of tissue culture wells (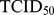) was determined by interpolation using the method of Reed and Muench [66] using serial dilutions of virus on Madin-Darby canine kidney (MDCK) cells. Colony forming units were counted for serial dilutions of bacteria on tryptic soy-agar plates supplemented with 3% (vol/vol) sheep erythrocytes. For infection experiments, virus was diluted in sterile PBS and administered at a dose of 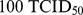 intranasally to groups of 6–10 mice lightly anesthetized with 2.5% inhaled isoflurane (Baxter, Deerfield, IL) in a total volume of 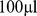 (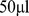 per nostril). On day 7 of the influenza infection, *S. pneumoniae* was diluted in sterile PBS and administered at a dose of 100 CFU or 1000 CFU intranasally to mice lightly anesthetized with 2.5% inhaled isoflurane (Baxter, Deerfield, IL) in a total volume of 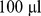 (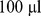 per nostril). Mice were weighed at the onset of infection and each subsequent day for illness and mortality. Mice were euthanized if they became moribund or lost 30% of their starting body weight.

### Lung titers

Mice were euthanized by 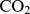 asphyxiation. Lungs were aseptically harvested, washed three times in PBS, and placed in 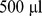 sterile PBS. Lungs were mechanically homogenized using the Ultra-Turrax T8 homogenizer (IKA-werke, Staufen, Germany). Lung homogenates were pelleted at 10,000 rpm for 5 minutes and the supernatants were used to determine the viral and bacterial titers for each set of lungs using serial dilutions on MDCK monolayers and on tryptic soy-agar plates supplemented with 3% (vol/vol) sheep erythrocytes, respectively.

### Mathematical models

#### Influenza A virus infection

We consider a target cell limited model that incorporates an eclipse phase, originally presented in Baccam et al. (2006) [67], to describe IAV kinetics. Although a number of models for influenza exist (reviewed in [38], [40]), we chose this model to analyze the viral titer data because of its simplicity and its proven ability to estimate parameters from viral titer data, especially in the context of murine infection systems in which the influenza viruses PR8 and PR8-PB1-F2(1918) were used [41]. This model depicts an influenza infection using four populations: susceptible epithelial (target) cells (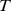), two sets of infected cells (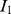 and 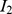), and free virus (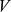). Target cells become infected at a rate 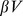 per cell. Newly infected cells (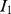) enter an eclipse phase before virion production begins. This period tends to be rather short, e.g., 4–6 hours, and for simplicity we assume no cell death occurs during this period. Cells, 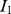, transition to productively infected cells (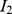) at a rate 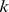 per cell. Productively infected cells are lost (e.g., by apoptosis, by viral cytopathic effects or by removal by immune cells) at a rate 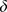 per cell. The average total infected cell lifetime is 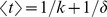. Virus production occurs at a rate 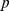 per cell, and virions are cleared at a rate 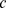 (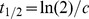 is the viral half-life). The following equations represent these dynamics.
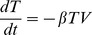
(1)

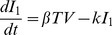
(2)

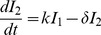
(3)

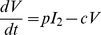
(4)


Data and models represent only infectious virus. Noninfectious virus is not detected by the experimental assay used and is not included in the model. This model does not specify mechanisms for some processes. For example, 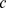 and 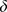 encompass both viral effects and immune mechanisms. It is thus possible that some of the parameters change with time. Here, we assume that all parameters are constant and use previously established parameter values that fit the observed viral titer data in the absence of a bacterial infection.

#### *Streptococcus pneumoniae* infection

To describe a pneumococcal lung infection in the absence of an antecedent viral infection, we use a model of the initial interaction between pneumococci and the first arm of the immune system, alveolar macrophages (AMs) [8]. We chose this model to analyze the bacterial titer data because it represents the simplest biologically relevant model, which allows for parameter estimation given the amount of data, and has the ability to match initial bacterial titer data from mice infected with pneumococal strain D39 [8].

The model we use considers two populations corresponding to pneumococci (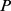) and alveolar macrophages (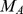). Pneumococci proliferate logistically at a maximum rate 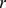 with a tissue carrying capacity of 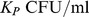. Phagocytosis of free bacteria occurs at rate 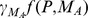 per cell. This rate decreases with pneumococal population size according to the function 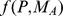,
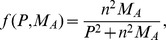
where 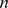 is the maximum number of bacteria phagocytosed per alveolar macrophage. AMs enter the interstial space at constant rate 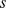 and are removed at rate 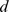. We take these cells to be in quasi-steady state such that 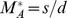. This reduces the model to a single differential equation for the pneumococcal population,
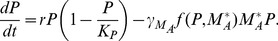
(5)


We again assume that all parameters are constant and use previously established parameter values that fit the observed bacterial titer data in the absence of a viral infection [8]. The units of the initial value (CFU per ml of lung homogenate) differs from the units of initial inocula (CFU) used in the experiments. We assume only a portion of bacteria reach the lungs since some bacteria could be quickly trapped in the airway and removed by mucocilliary mechanisms. Therefore, the initial value of pneumococci (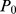) is chosen as one log lower than the inoculum size (e.g., for an inoculum of 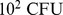, 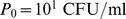).

#### Coinfection model

We developed a kinetic model that couples Equations (1)–(4) with Equation (5) based on proposed mechanisms of interaction between influenza and pneumococcus. We consider two viral effects that may enhance the secondary bacterial infection: increased bacterial adherence to epithelial cells and alveolar macrophage dysfunction. We also consider one potential bacterial effect that may enhance the viral coinfection: increased viral release from infected epithelial cells. Altering other processes in the model, such as the rates of viral infectivity (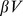) or viral clearance (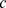), produced smaller effects on model dynamics.

#### ***Increased bacterial adherence to epithelial cells***

Acquisition of a secondary bacterial infection following influenza is, at least partially, a consequence of increased adherence of pneumococci to epithelial cells infected with influenza [17], [68]. The sialidase activity of viral NA may work in concert with or replace pneumococcal NA to expose viable receptors that pneumococci attach to [5], [17], [69], [70]. We translate these empirical findings into a mathematical description by assuming that an increase of available infection sites (i.e., improved pneumococcal adherence) results in an elevated bacterial carrying capacity (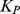). In our model, the increase occurs proportional to free virus density with constant of proportionality 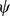.

#### ***Increased epithelial cell death from bacterial adherence***

Pneumococci kill host epithelial cells with the toxin pneumolysin, which lyses cells by creating pores in cellular membranes during attachment [71]. An increased attachment rate of pneumococci to cells infected with influenza has been observed [17], [68] and suggests that the death rate of these cells may increase in the presence of pneumococci. In our model, death of infected epithelial cells (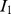, 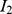) from bacterial attachment occurs at a rate 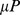. This toxic effect may also modify target cell (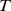) dynamics; however, 7 days after influenza inoculation the target cell population is near zero. Without inclusion of cell regeneration, this term has negligible effects on the coinfection dynamics.

#### ***Decreased rate of phagocytosis by alveolar macrophages***

Alveolar macrophages have a protective role in pneumococcal infections by providing initial clearance and modulation of the inflammatory response [26], [27], [72]. An influenza infection may modify this response and suppress innate protection against bacterial pathogens [23], [25]. The inability of alveolar macrophages to phagocytose incoming pneumococci could facilitate bacterial establishment and growth. We include this reduction in bacterial clearance in our model as a saturating function of viral presence: 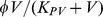, where 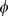 is the maximal reduction of the phagocytosis rate and 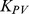 is the half-saturation constant.

#### ***Increased virion release from infected epithelial cells***

The underlying process that results in viral titer rebound following pneumococcal challenge is unknown. One plausible hypothesis is the interaction of viral and bacterial neuraminidase. Influenza NA promotes the release of virions from infected cells [73], and presence of bacterial NA may enhance this process, although other processes may also be involved. We use the function 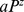, where 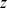 is between 0 and 1, to incorporate bacterial promotion of viral production and release from productively infected cells. We chose this function rather than a Hill-type function because it has fewer parameters and has a more gradual effect rather than a quickly saturating effect.

Together, these dynamics are represented in Figure 3 and described by Equations (6)–(10), where the viral and bacterial interactions are highlighted in bold.
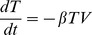
(6)

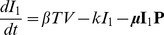
(7)

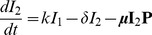
(8)

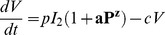
(9)

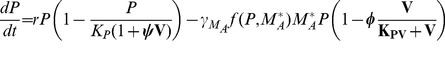
(10)


### Model parameters

We use the parameter values for each model of single pathogen infections (i.e., influenza (Equations (1)–(4)) and pneumococcus (Equation (5))) that were established by our earlier work [8], [41]. The best-fit parameter estimates from these earlier studies are provided in Table 1. We use the two largest data sets (i.e., PR8 or PR8-PB1-F2(1918) infection followed 7 days later with 1000 CFU D39 infection) to fit Equations (6)–(10) simultaneously to the lung viral and bacterial titers. We assume errors in the 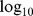 titer values are normally distributed. To account for unequal viral and bacterial measurements, we use a cost function that weighted the viral and bacterial data equally, i.e., the cost 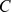 for a parameter set 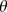 was 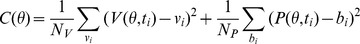. Here, 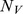 and 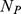 are the number of viral data points 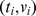 and bacterial data points 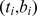, respectively, and 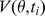 and 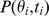 are the corresponding model predictions. When the number of viral and bacterial measurements are equal, minimizing 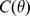 is equivalent to minimizing the negative log-likelihood. The cost is minimized across parameter regimes using the Matlab minimization subroutine (*fmincon*) and ODE solver (*ode45*) to compare experimental and predicted values of 
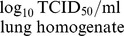
 and 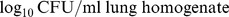.

To explore and visualize the regions of parameter space consistent with the model and data, we use a Bayesian ensemble method [43] with a uniform prior on the logs of the parameters (details in Text S1). For each parameter, we provide a 95% confidence interval (CI) computed from the ensemble. These calculations were performed with the software package SloppyCell [74], [75]. To assess the exclusion of individual model parameters, we compared the fit quality of the model using the small sample size corrected Akaike's Information Criteria (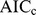) [76]:
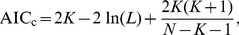
(11)where 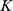 is the number of model parameters, 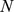 is the sample size, and 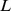 is the maximum likelihood value. A model with a lower 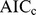 is considered to be a better model.

## Supporting Information

Text S1 Analysis of the coinfection model dynamics and individual parameters through a Bayesian ensemble analysis and a sensitivity analysis.
(PDF)
