# Supplementary material for: Kinetics of Coinfection with Influenza A Virus and Streptococcus pneumoniae
Source: PLoS Pathog. 2013 Mar 21;9(3):e1003238. doi: 10.1371/journal.ppat.1003238 (PMC3605146; doi:10.1371/journal.ppat.1003238)
Supplement: Text S1 — Analysis of the coinfection model dynamics and individual parameters through a Bayesian ensemble analysis and a sensitivity analysis. (PDF) [file ppat.1003238.s001.pdf]

Supporting Information

Kinetics of Coinfection with Influenza A Virus and *Streptococcus pneumoniae*
Amber M. Smith[1,*], Frederick R. Adler[2], Ruy M. Ribeiro[3,4], Ryan N. Gutenkunst[5], Julie L. McAuley[6], Jonathan A. McCullers[1], Alan S. Perelson[3]

[1]Department of Infectious Diseases, St. Jude Children's Research Hospital, Memphis, TN 38105, USA
[2]Departments of Mathematics and Biology, University of Utah, Salt Lake City, UT 84112, USA
[3]Theoretical Biology and Biophysics, Los Alamos National Laboratory, Los Alamos, NM 87545, USA
[4]Instituto de Medicina Molecular, Faculdade de Medicina da Universidade de Lisboa, Portugal
[5]Department of Molecular and Cellular Biology, University of Arizona, Tucson, AZ 85721, USA
[6]Department of Immunology and Microbiology, University of Melbourne, Victoria, Australia
[*]Email: amber.smith@stjude.org

Analysis of Model Parameters

To gain a better understanding of the coinfection model dynamics and reveal relationships between model solutions and parameters, we analyzed our model output and individual parameters through a Bayesian ensemble analysis and a sensitivity analysis.

Bayesian Ensemble Analysis

To examine the regions of parameter space consistent with the model and data, we used a Bayesian ensemble (Brown and Sethna (2003)) analysis with a uniform prior on the logs of all parameters. We placed bounds on the parameters to constrain them to reasonable values. Since biological estimates are not available for these parameters, we specified large ranges. We allowed the proportionality constant for the bacterial carrying capacity, ψ, to vary between 0 $(\text{TCID}_{50}/\text{ml})^{-1}$ and 1×10^{-7} $(\text{TCID}_{50}/\text{ml})^{-1}$, the maximal reduction in bacterial phagocytosis, ϕ, to vary between 0 and 1, the half-saturation constant, K_{PV}, to vary between 1×10^{2} $\text{TCID}_{50}/\text{ml}$ and 1×10^{5} $\text{TCID}_{50}/\text{ml}$, the rate of infected cell death from bacterial attachment, μ, to vary between 0 $(\text{CFU/ml})^{-1}$ and 1×10^{-8} $(\text{CFU/ml})^{-1}$, the rate of bacterial-induced viral release, a, to vary between 1×10^{-4} $(\text{CFU/ml})^{-z}$ and 5×10^{-1} $(\text{CFU/ml})^{-z}$, and the nonlinearity of the viral release effect, z, to vary between 0 and 1. These calculations were performed with the software package SloppyCell (Myers et

al. (2007), Gutenkunst et al. (2007)).

We performed the analysis for the two data sets we fit, i.e., infection with 1000 CFU D39 7 days after infection with either (i) PR8 or (ii) PR8-PB1-F2(1918), and found consistent behavior among the parameters. In Figures S1-S2, we show the distributions of parameter values and the behavior of parameters in the form of two-parameter projections ("ensembles") for PR8 and PR8-PB1F2(1918) coinfection, respectively. Importantly, we find that ϕ (alveolar macrophage impairment) is tightly constrained and large ranges for two parameters, μ and ψ, with values that approach the lower boundary (i.e., 0). This suggests that the two effects determined by μ (increased infected cell death) and ψ (increased bacterial carrying capacity) have minimal impact on the model outcome compared to the other two effects, i.e., alveolar macrophage impairment ($\phi V/(K_{PV} + V)$) and increased viral production/release (aP^z). We confirmed this by fitting the model with $\mu, \psi = 0$ (Figures S3-S4) and found no appreciable differences in the parameters or confidence intervals (Table S1) or in the model dynamics (not shown). Not surprisingly, these results also show a strong correlation between the rate of bacterial-induced viral production/release (a) and the nonlinearity of this release (z) and between the decrease in bacterial phagocytosis (ϕ) and the half-saturation constant (K_{PV}). However, an important point from these bivariate plots is that we do not find correlations among the effects/parameters. This indicates that there are no substantial trade-offs among the fitted parameters.

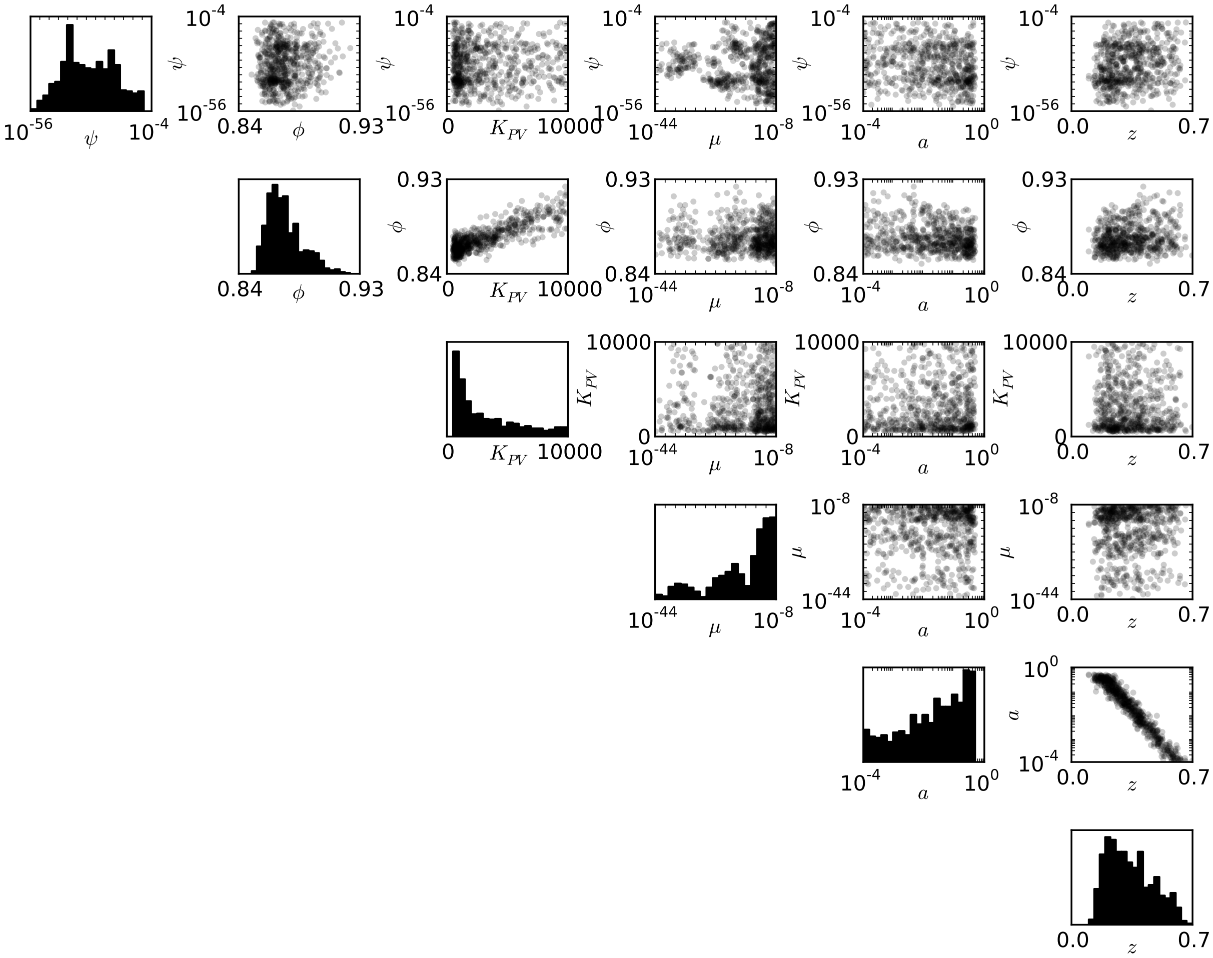

Figure S1: Bayesian ensemble analysis of the coinfection model for coinfection with PR8 and 1000 CFU D39. Parameter distributions and ensembles, in the form of two parameter projections of each fit, from the Bayesian ensemble analysis of the coinfection model (Equations (6)-(10)) and lung titers from mice infected with 100 $TCID_{50}$ PR8 virus followed 7 days later by 1000 CFU *S. pneumoniae* strain D39.

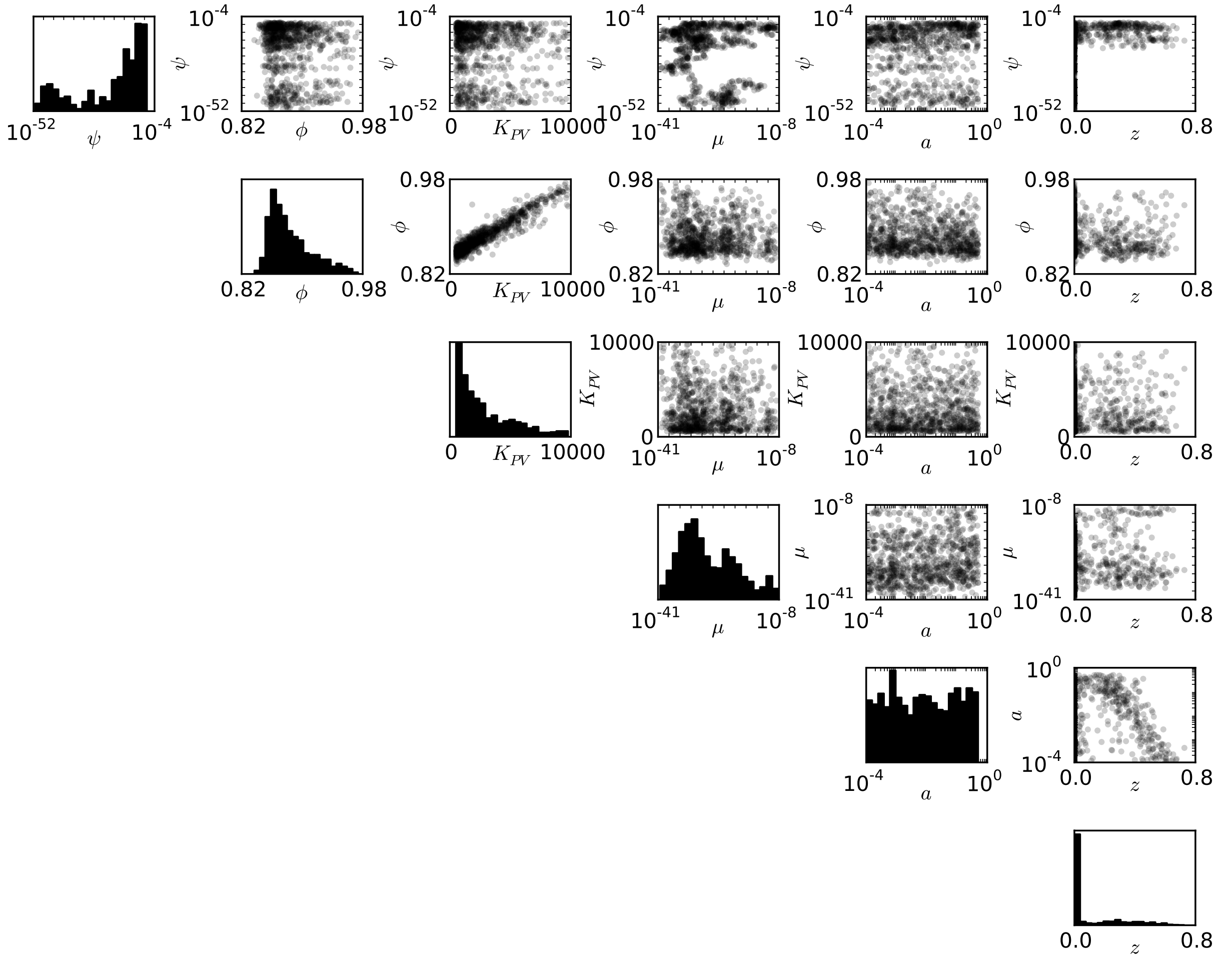

Figure S2: Bayesian ensemble analysis of the coinfection model for coinfection with PR8-PB1-F2(1918) and 1000 CFU D39. Parameter distributions and ensembles, in the form of two parameter projections of each fit, from the Bayesian ensemble analysis of the coinfection model (Equations (6)-(10)) and lung titers from mice infected with 100 $TCID_{50}$ PR8-PB1-F2(1918) virus followed 7 days later by 1000 CFU *S. pneumoniae* strain D39.

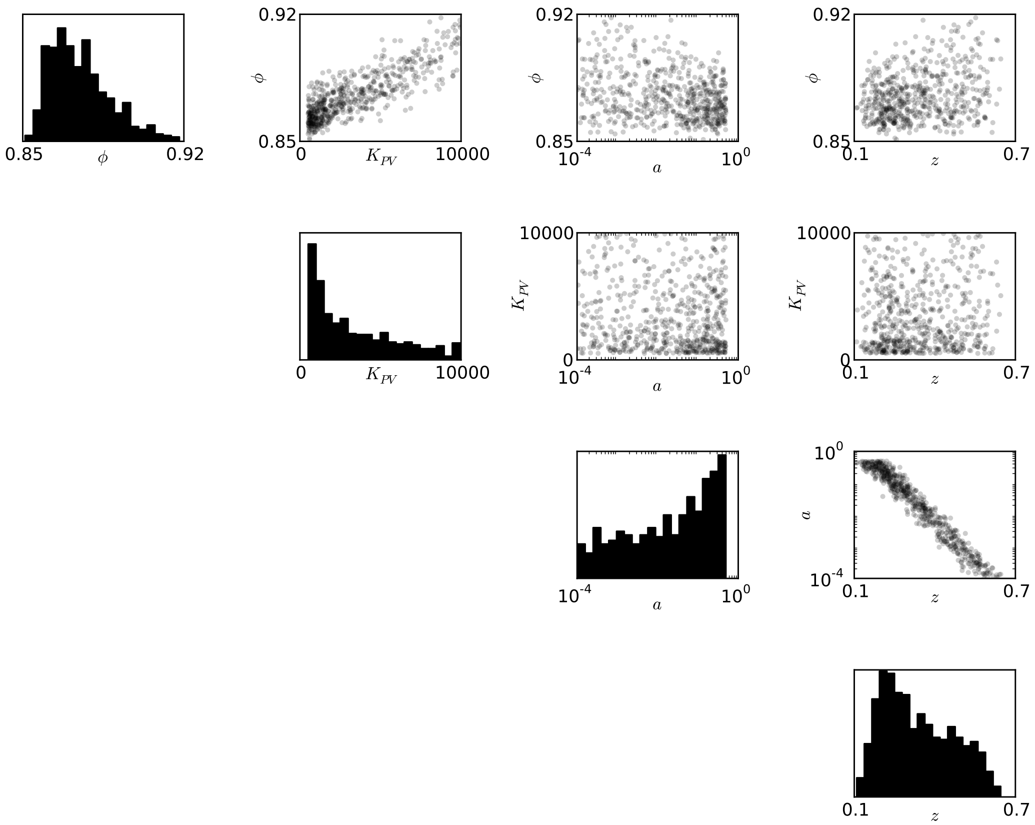

Figure S3: Bayesian ensemble analysis of the coinfection model with $\mu = 0$ and $\psi = 0$ for coinfection with PR8 and 1000 CFU D39. Parameter distributions and ensembles, in the form of two parameter projections of each fit, from the Bayesian ensemble analysis of the coinfection model (Equations (6)-(10)) with $\mu = 0$ and $\psi = 0$ and lung titers from mice infected with 100 $TCID_{50}$ PR8 virus followed 7 days later by 1000 CFU *S. pneumoniae* strain D39.

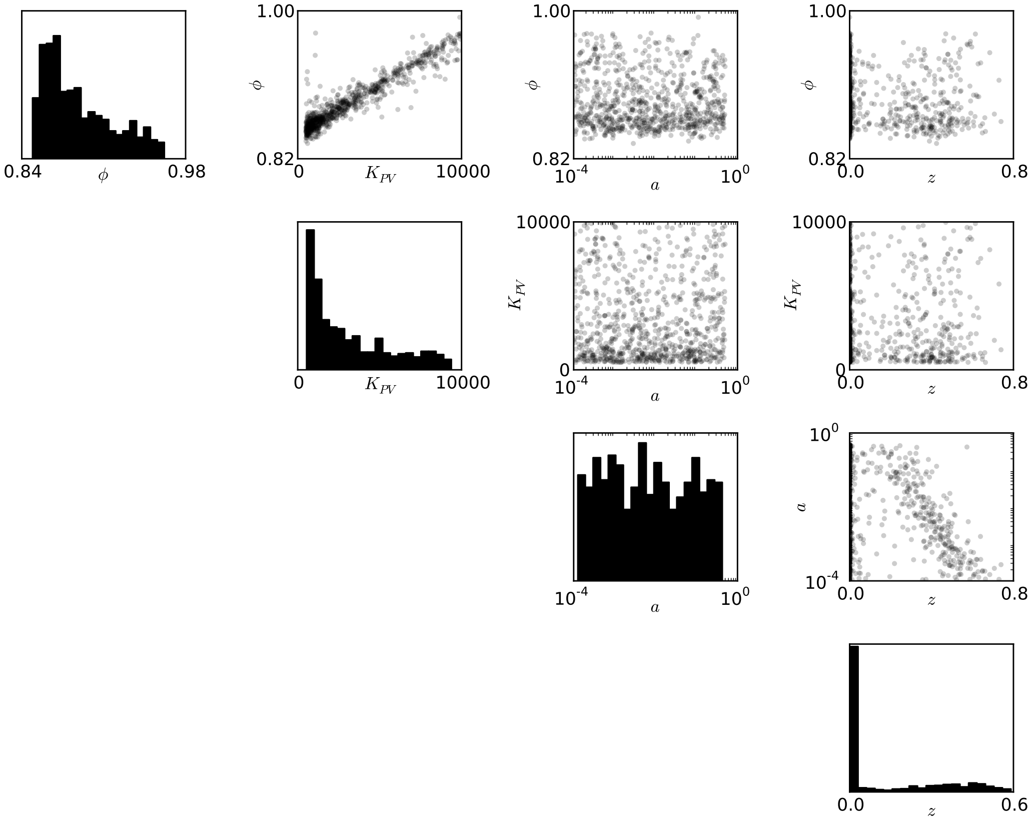

Figure S4: Bayesian ensemble analysis of the coinfection model with $\mu = 0$ and $\psi = 0$ for coinfection with PR8-PB1-F2(1918) and 1000 CFU D39. Parameter distributions and ensembles, in the form of two parameter projections of each fit, from the Bayesian ensemble analysis of the coinfection model (Equations (6)-(10)) with $\mu = 0$ and $\psi = 0$ and lung titers from mice infected with 100 $TCID_{50}$ PR8-PB1-F2(1918) virus followed 7 days later by 1000 CFU *S. pneumoniae* strain D39.

Table S1: Parameter estimates and 95% confidence intervals from the coinfection model (Equations (6)-(10)) with $\mu = 0$ and $\psi = 0$ for the dynamics of infection with 1000 CFU D39 7 days after PR8 or PR8-PB1-F2(1918) infection.

Parameter	Description (Units)	Value	
**Viral Effects on Bacteria**			
		**PR8**	**PR8-PB1-F2(1918)**
ϕ	Decrease in phagocytosis rate	0.87 [0.86, 0.91]	0.85 [0.85, 0.96]
K_{PV}	Half-saturation constant ($TCID_{50}$/ml)	1.8×10^3 [5.5×10^2, 9.5×10^3]	1.8×10^3 [5.4×10^2, 9.4×10^3]
**Bacterial Effects on Virus**			
a	Increase in virion production/release ((CFU/ml)$^{-z}$)	1.2×10^{-3} [1.5×10^{-4}, 4.6×10^{-1}]	1.8×10^{-1} [1.3×10^{-3}, 4.3×10^{-1}]
z	Nonlinearity of virion production/release	0.48 [0.14, 0.59]	0.29 [0, 0.59]

*Because of the dependency between some parameters, there are many sets of parameters that give rise to equivalent fits.

Individual Parameter Perturbations

We then analyzed the output of our model in response to changes in the parameters by simulating Equations (6)-(10) (in the main text) over a range of each of the six coinfection model parameters ($q = \mu$, z, a, ψ, ϕ, K_{PV}) such that the maximum and minimum values were set to ±50% of its value in Table 2, respectively. The effect that each parameter has on the viral (panels (a)) and bacterial (panels (b)) dynamics are shown for pneumoccocal initial value $P_0 = 10^2$ CFU/ml for preinfection with PR8 in Figures S5-S10 and for preinfection with PR8-PB1-F2(1918) in Figure S11-S14. There is no significant difference for PR8 preinfection with the lower initial condition, $P_0 = 10$ CFU/ml (not shown).

These analyses suggest that small perturbations in a (increase in viral production/release), z (nonlinearity in virion production/release), ϕ (decrease in phagocytosis rate), K_{PV} (half-saturation constant, only with preinfection with PR8-PB1-F2(1918)) results in large effects on viral and bacterial levels, while perturbations in the other model parameters (μ and ψ) have little effect. In addition, as the value of ϕ (and K_{PV} in the case of PR8-PB1F2(1918)) decreases, a critical threshold exists such that the bacterial population is significantly decreased or nonexistent.

Preinfection with PR8

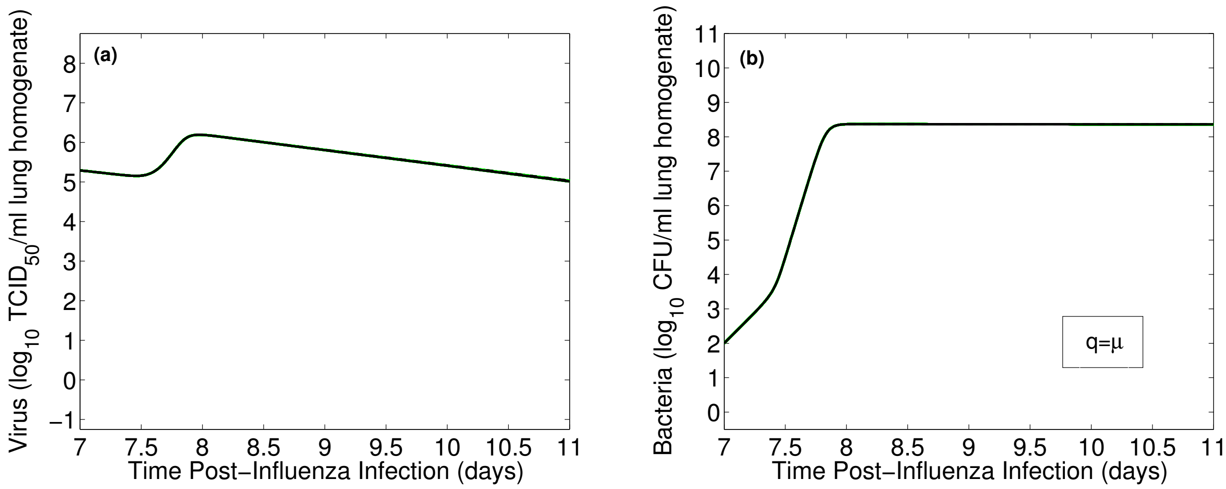

Figure S5: Perturbation of μ in the coinfection model for infection with PR8 and 1000 CFU D39. Simulation results for virus (panel (a)) and bacteria (panel (b)) for perturbations in μ (rate of infected cell death from bacterial adherence) with $P_0 = 10^2$ CFU/ml and PR8 preinfection. The solid and dashed black lines represents a $\pm 50\%$ increase/decrease from the value in Table 2 (5.2×10^{-10} (CFU/ml)$^{-1}$), respectively, and the green lines represent intermediate values. Here, the lines overlap since no visible changes occur in the model dynamics with changes in μ.

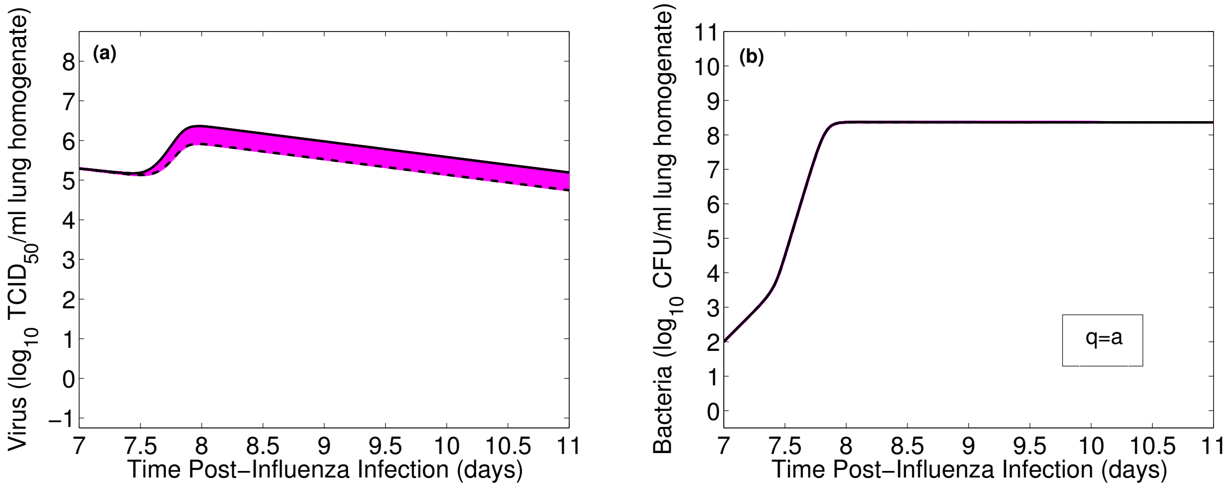

Figure S6: Perturbation of a in the coinfection model for infection with PR8 and 1000 CFU D39. Simulation results for virus (panel (a)) and bacteria (panel (b)) for perturbations in a (rate of enhanced virion release from infected cells in the presence of bacteria) with $P_0 = 10^2$ CFU/ml and PR8 preinfection. The solid and dashed black lines represents a $\pm 50\%$ increase/decrease from the value in Table 2 (1.3×10^{-3} (CFU/ml)$^{-z}$), respectively, and the magenta lines represent intermediate values.

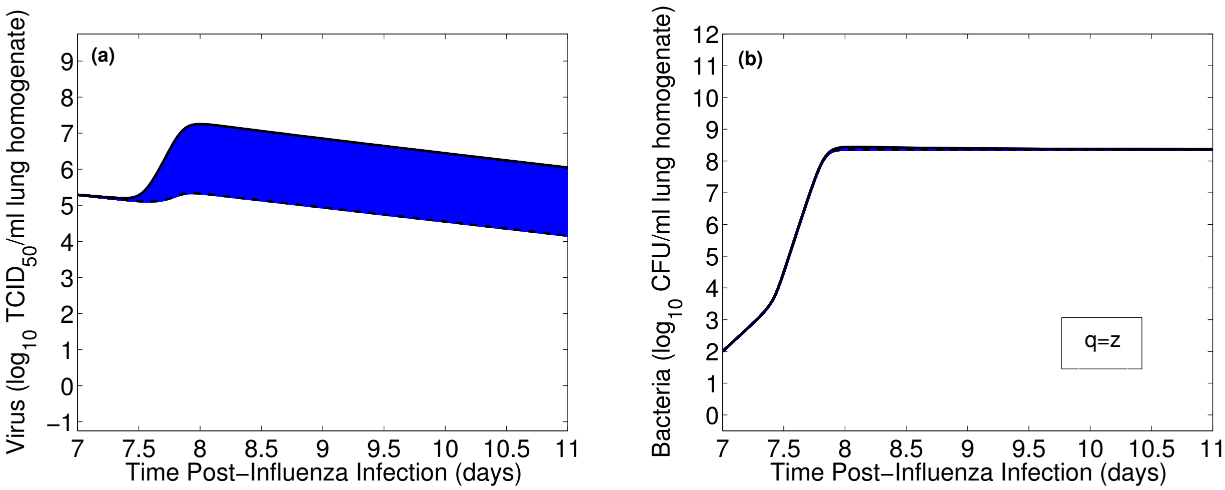

Figure S7: Perturbation of z in the coinfection model for infection with PR8 and 1000 CFU D39. Simulation results for virus (panel (a)) and bacteria (panel (b)) for perturbations in z (nonlinearity of the enhancement of virion release from infected cells in the presence of bacteria) with $P_0 = 10^2$ CFU/ml and PR8 preinfection. The solid and dashed black lines represents a $\pm 50\%$ increase/decrease from the value in Table 2 (0.50), respectively, and the blue lines represent intermediate values.

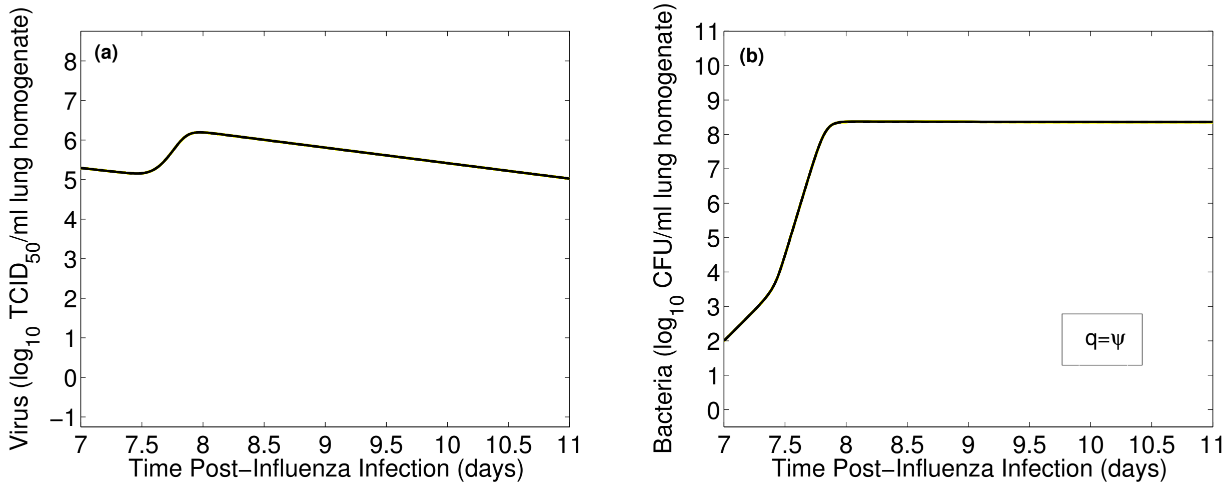

Figure S8: Perturbation of ψ in the coinfection model for infection with PR8 and 1000 CFU D39. Simulation results for virus (panel (a)) and bacteria (panel (b)) for perturbations in ψ (rate of the change in bacterial carrying capacity in the presence of virus) with $P_0 = 10^2$ CFU/ml and PR8 preinfection. The solid and dashed black lines represents a $\pm 50\%$ increase/decrease from the value in Table 2 (1.2×10^{-8} $(\text{TCID}_{50}/\text{ml})^{-1}$), respectively, and the yellow lines represent intermediate values. Here, the lines overlap since no visible changes occur in the model dynamics with changes in ψ.

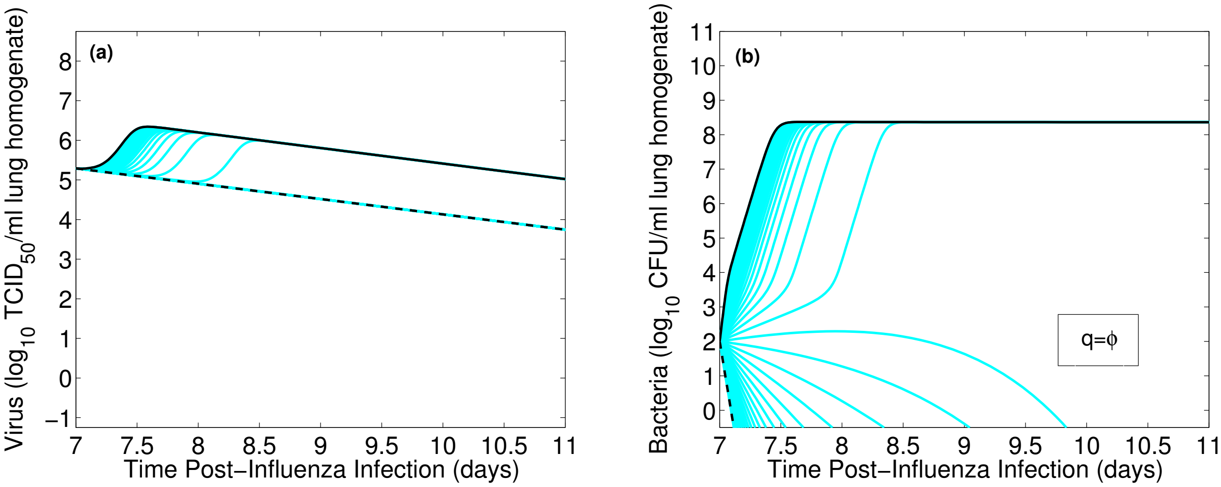

Figure S9: Perturbation of ϕ in the coinfection model for infection with PR8 and 1000 CFU D39. Simulation results for virus (panel (a)) and bacteria (panel (b)) for perturbations in ϕ (rate of bacterial phagocytosis in the presence of virus) with $P_0 = 10^2$ CFU/ml and PR8 preinfection. The solid and dashed black lines represents a ±50% increase/decrease from the value in Table 2 (0.87), respectively, and the cyan lines represent intermediate values.

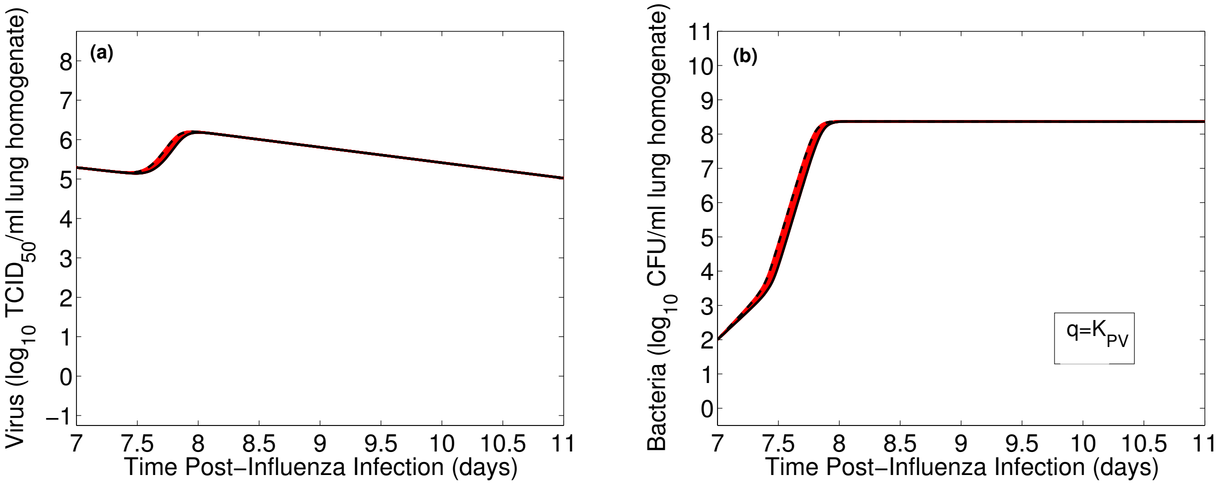

Figure S10: Perturbation of K_{PV} in the coinfection model for infection with PR8 and 1000 CFU D39. Simulation results for virus (panel (a)) and bacteria (panel (b)) for perturbations in K_{PV} (half-saturation constant of the effect on bacterial phagocytosis in the presence of virus) with $P_0 = 10^2$ CFU/ml and PR8 preinfection. The solid and dashed black lines represents a ±50% increase/decrease from the value in Table 2 (1.8×10^3 $TCID_{50}$/ml), respectively, and the red lines represent intermediate values.

Preinfection with PR8-PB1-F2(1918)

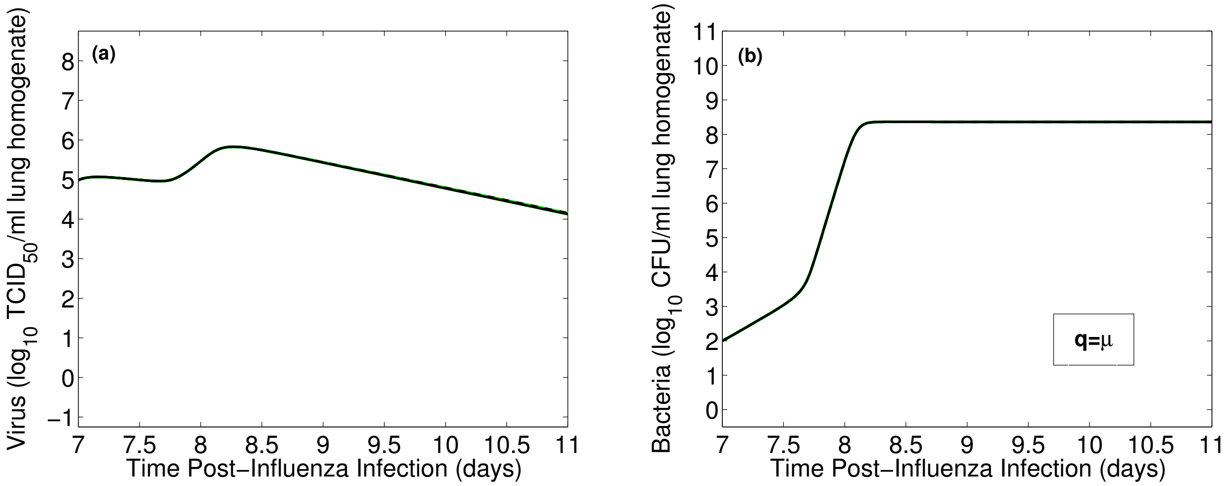

Figure S11: Perturbation of μ in the coinfection model for infection with PR8-PB1-F2(1918) and 1000 CFU D39. Simulation results for virus (panel (a)) and bacteria (panel (b)) for perturbations in μ (rate of infected cell death from bacterial adherence) with $P_0 = 10^2$ CFU/ml and PR8-PB1-F2(1918) preinfection. The solid and dashed black lines represents a $\pm 50\%$ increase/decrease from the value in Table 2 (8.9×10^{-10} (CFU/ml)$^{-1}$), respectively, and the green lines represent intermediate values. Here, the lines overlap since no visible changes occur in the model dynamics with changes in μ.

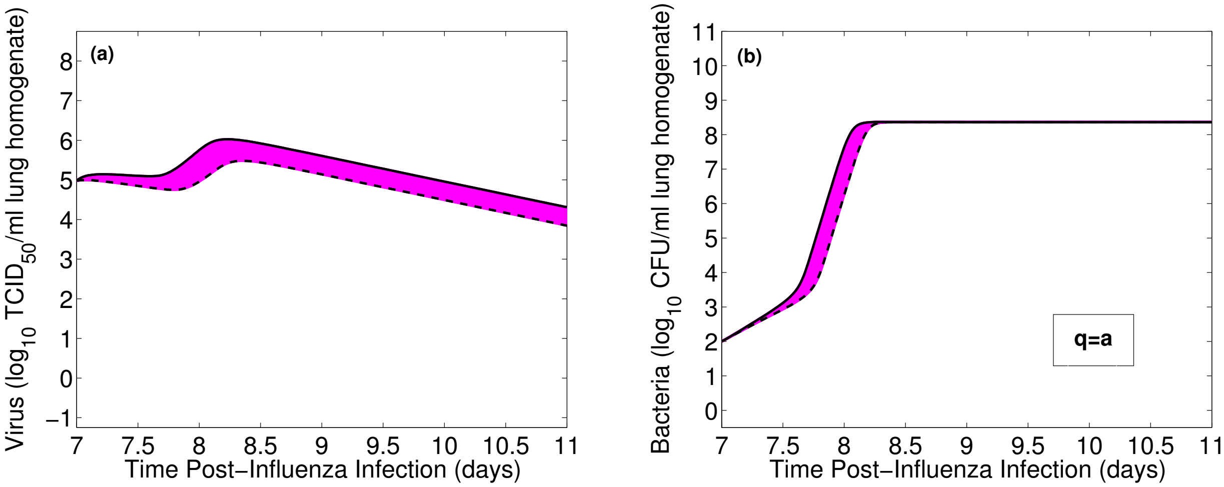

Figure S12: Perturbation of a in the coinfection model for infection with PR8-PB1-F2(1918) and 1000 CFU D39. Simulation results for virus (panel (a)) and bacteria (panel (b)) for perturbations in a (rate of enhanced virion release from infected cells in the presence of bacteria) with $P_0 = 10^2$ CFU/ml and PR8-PB1-F2(1918) preinfection. The solid and dashed black lines represents a $\pm 50\%$ increase/decrease from the value in Table 2 (1.7×10^{-1} (CFU/ml)$^{-z}$), respectively, and the magenta lines represent intermediate values.

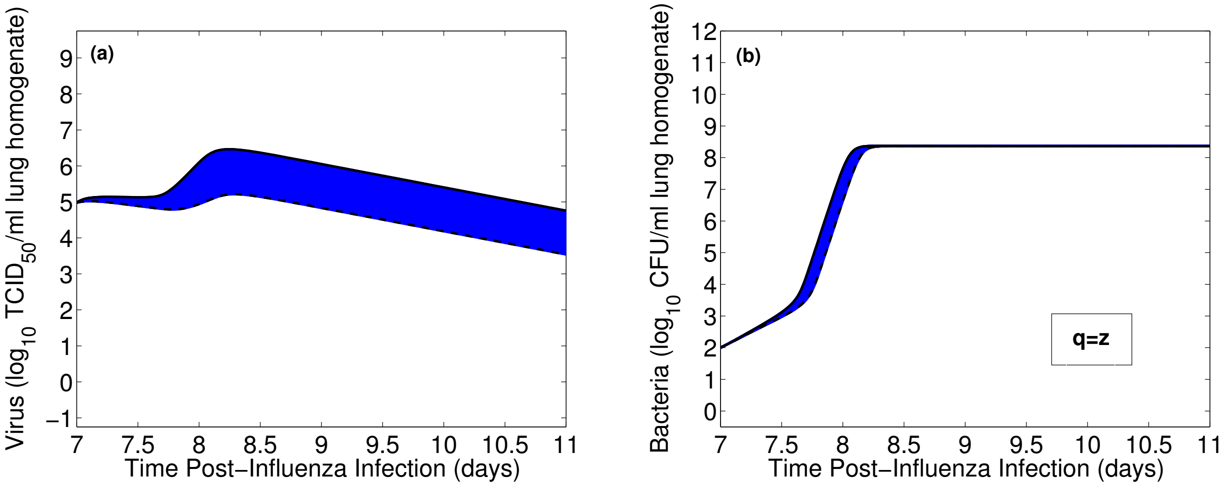

Figure S13: Perturbation of z in the coinfection model for infection with PR8-PB1-F2(1918) and 1000 CFU D39. Simulation results for virus (panel (a)) and bacteria (panel (b)) for perturbations in z (nonlinearity of the enhancement of virion release from infected cells in the presence of bacteria) with $P_0 = 10^2$ CFU/ml and PR8-PB1-F2(1918) preinfection. The solid and dashed black lines represents a ±50% increase/decrease from the value in Table 2 (0.30), respectively, and the blue lines represent intermediate values.

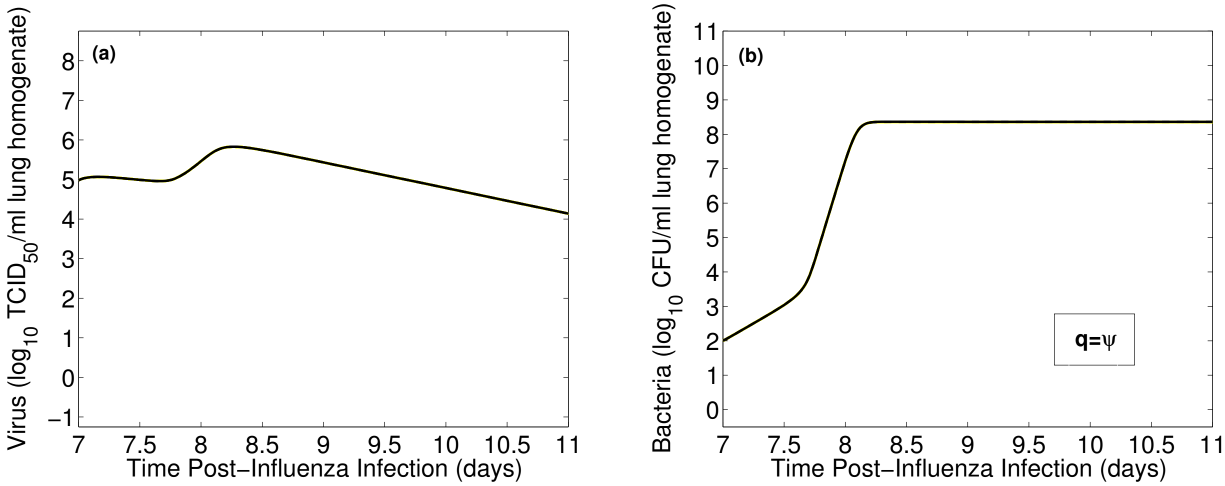

Figure S14: Perturbation of ψ in the coinfection model for infection with PR8-PB1-F2(1918) and 1000 CFU D39. Simulation results for virus (panel (a)) and bacteria (panel (b)) for perturbations in ψ (rate of the change in bacterial carrying capacity in the presence of virus) with $P_0 = 10^2$ CFU/ml and PR8-PB1-F2(1918) preinfection. The solid and dashed black lines represents a ±50% increase/decrease from the value in Table 2 (8.9×10^{-9} $(TCID_{50}/ml)^{-1}$), respectively, and the yellow lines represent intermediate values. Here, the lines overlap since no visible changes occur in the model dynamics with changes in ψ.

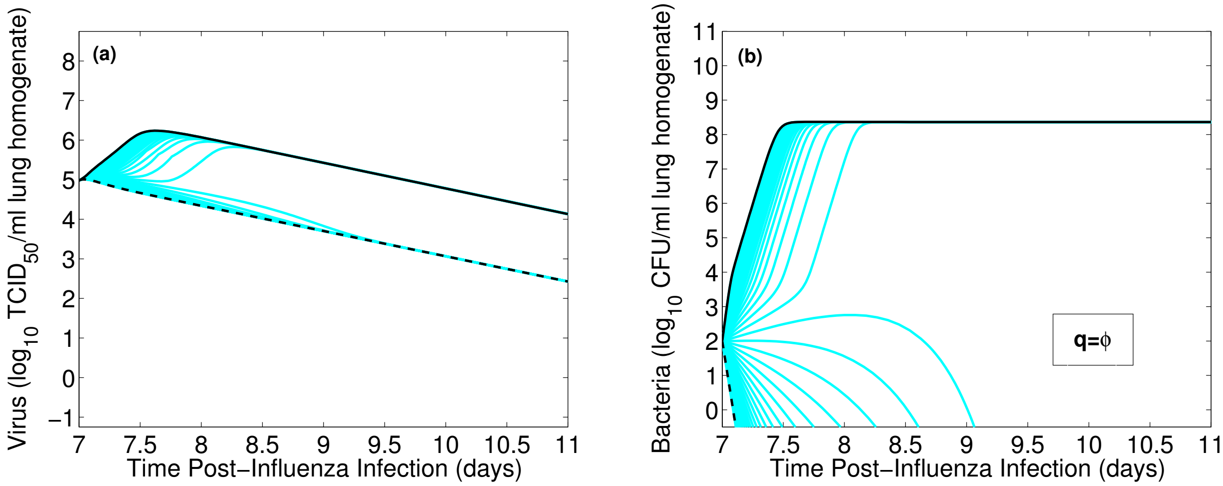

Figure S15: Perturbation of ϕ in the coinfection model for infection with PR8-PB1-F2(1918) and 1000 CFU D39. Simulation results for virus (panel (a)) and bacteria (panel (b)) for perturbations in ϕ (rate of bacterial phagocytosis inhibition in the presence of virus) with $P_0 = 10^2$ CFU/ml and PR8-PB1-F2(1918) preinfection. The solid and dashed black lines represents a ±50% increase/decrease from the value in Table 2 (0.85), respectively, and the cyan lines represent intermediate values.

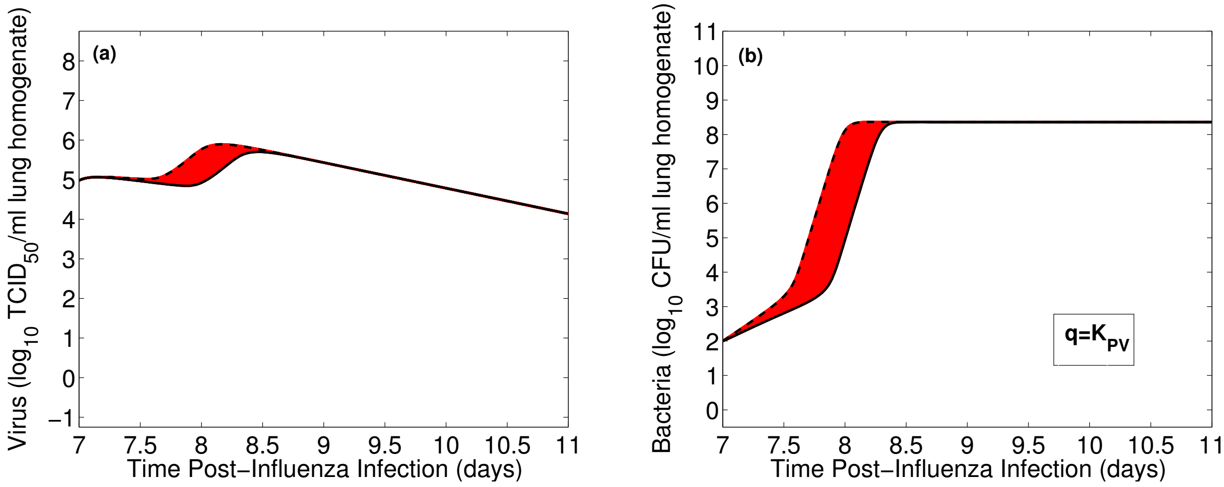

Figure S16: Perturbation of K_{PV} in the coinfection model for infection with PR8-PB1-F2(1918) and 1000 CFU D39. Simulation results for virus (panel (a)) and bacteria (panel (b)) for perturbations in K_{PV} (half-saturation constant of the effect on bacterial phagocytosis in the presence of virus) with $P_0 = 10^2$ CFU/ml and PR8-PB1-F2(1918) preinfection. The solid and dashed black lines represents a ±50% increase/decrease from the value in Table 2 (1.8×10^3 $TCID_{50}$/ml), respectively, and the red lines represent intermediate values.

Differential Sensitivity Analysis

We also performed a more rigorous differential sensitivity analysis of the coinfection model parameters using a method known as the direct method (Eslami, 1994, Frank, 1978). For the coinfection model (Equations (6)-(10) in the main text), the sensitivity functions (**S**) for any parameter q_i are defined as

$$\begin{aligned}
S_1: &\quad T_{q_i}(t) = \frac{\partial}{\partial q_i} T(t, \mathbf{q}) \\
S_2: &\quad I_{1,q_i}(t) = \frac{\partial}{\partial q_i} I_1(t, \mathbf{q}) \\
S_3: &\quad I_{2,q_i}(t) = \frac{\partial}{\partial q_i} I_2(t, \mathbf{q}) \\
S_4: &\quad V_{q_i}(t) = \frac{\partial}{\partial q_i} V(t, \mathbf{q}) \\
S_5: &\quad P_{q_i}(t) = \frac{\partial}{\partial q_i} P(t, \mathbf{q})
\end{aligned} \tag{S1}$$

The corresponding sensitivity equations, which interpret how changes in parameter values influence the model solution over time, are generated by differentiating the model equations with respect to each parameter:

$$\begin{aligned}
\frac{dS_1}{dt} &= -\beta\left(VS_1 + TS_4\right) + \frac{\partial \dot{T}}{\partial q_i} \\
\frac{dS_2}{dt} &= \beta\left(VS_1 + TS_4\right) - \left(k + \mu P\right) S_2 - \mu I_1 S_5 + \frac{\partial \dot{I}_1}{\partial q_i} \\
\frac{dS_3}{dt} &= kS_2 - \left(\delta + \mu P\right) S_3 - \mu I_2 S_5 + \frac{\partial \dot{I}_2}{\partial q_i} \\
\frac{dS_4}{dt} &= p\left(1 + aP^z\right) S_3 - cS_4 + pazI_2P^{z-1} S_5 + \frac{\partial \dot{V}}{\partial q_i} \\
\frac{dS_5}{dt} &= \left[\frac{\psi r P^2}{K_P(1+\psi V)^2} + \frac{\phi K_{PV} \gamma_{M_A} f(P, M_A^*) M_A^* P}{(K_{PV}+V)^2}\right] S_4 + r\left(1 - \frac{2P}{K_P(1+\psi V)}\right) S_5 \\
&\quad - \gamma_{M_A} M_A^* \left(1 - \frac{\phi}{K_{PV}+V}\right)\left(\frac{\partial f(P, M_A^*)}{\partial P} + f(P, M_A^*) P\right) S_5 + \frac{\partial \dot{P}}{\partial q_i}
\end{aligned} \tag{S2}$$

For each of the 6 model parameters of interest (μ, z, a, ψ, ϕ, K_{PV}), a set of these 5

equations are constructed and solved simultaneously with the model equations (Equations (6)-(10) in the main text) using *ode45* in Matlab. We analyze these equations using the logarithmic sensitivity solutions (e.g., $\phi P_\phi(t)/P(t)$), which are dimensionless and reflect the percentage change in the solution that occurs when a parameter is varied. The logarithmic sensitivity solution curves are presented in Figures S17-S19.

Each set of parameters is plotted separately for the two initial pneumococcal conditions ($P_0 = 10^2$ CFU/ml (Figure S17)and $P_0 = 10^1$ CFU/ml (Figure S18)) and for the preinfection with the PR8-PB1-F2(1918) virus (Figures S19). Figures S17-S19 represent the effect that positive perturbations in the model parameters have on solutions.

For example, an increase in the parameters ϕ (rate of bacterial phagocytosis inhibition) impacts both the viral and bacterial solutions positively (Figure S13a,b-15a,b insets). That is, for instance, increasing ϕ results in an increase in the bacterial population, with a 150% increase before 24 hours post-inoculation when $P_0 = 10^2$ CFU/ml. The effect of this parameter then decreases quickly and becomes negligible slightly after 24 hours post-inoculation.

Similarly, an increase in the two parameters that affect viral production in the presence of bacteria (a and z), results in an increase of both virus and bacteria, although these effects occur slightly later in the infection since these terms are dependent on bacterial growth. The effect of perturbing μ or ψ is minimal, which again highlights that the two dominating mechanisms at play are the increase of viral production and the alveolar macrophage impairment.

When interpreting the results in Figures S17-S19, the dynamics of the original model must be taken into consideration. For example when the bacterial challenge is given 7 days post-influenza inoculation, target cells and infected cells are in low numbers. Therefore, altering the value of the parameters has only minimal effects on the model solution for these variables (e.g., T, I_1) since, say, a 25% decrease in target cells at 24 hours post-inoculation when $T \approx 0$ (Figure S17c inset) is negligible. This is not the case, however, for the viral and bacterial populations. For these dynamics, we must interpret the sensitivity analysis

results throughout the entire length of the simulation. It is also important to note the scales in Figures S13-S15. Although the curves have similar dynamics when comparing initial bacterial conditions and viral preinfections, their scales vary. When comparing the results for each of the initial conditions, $P_0 = 10^2$ CFU/ml and $P_0 = 10^1$ CFU/ml, the effects are slightly more pronounced for the higher initial condition.

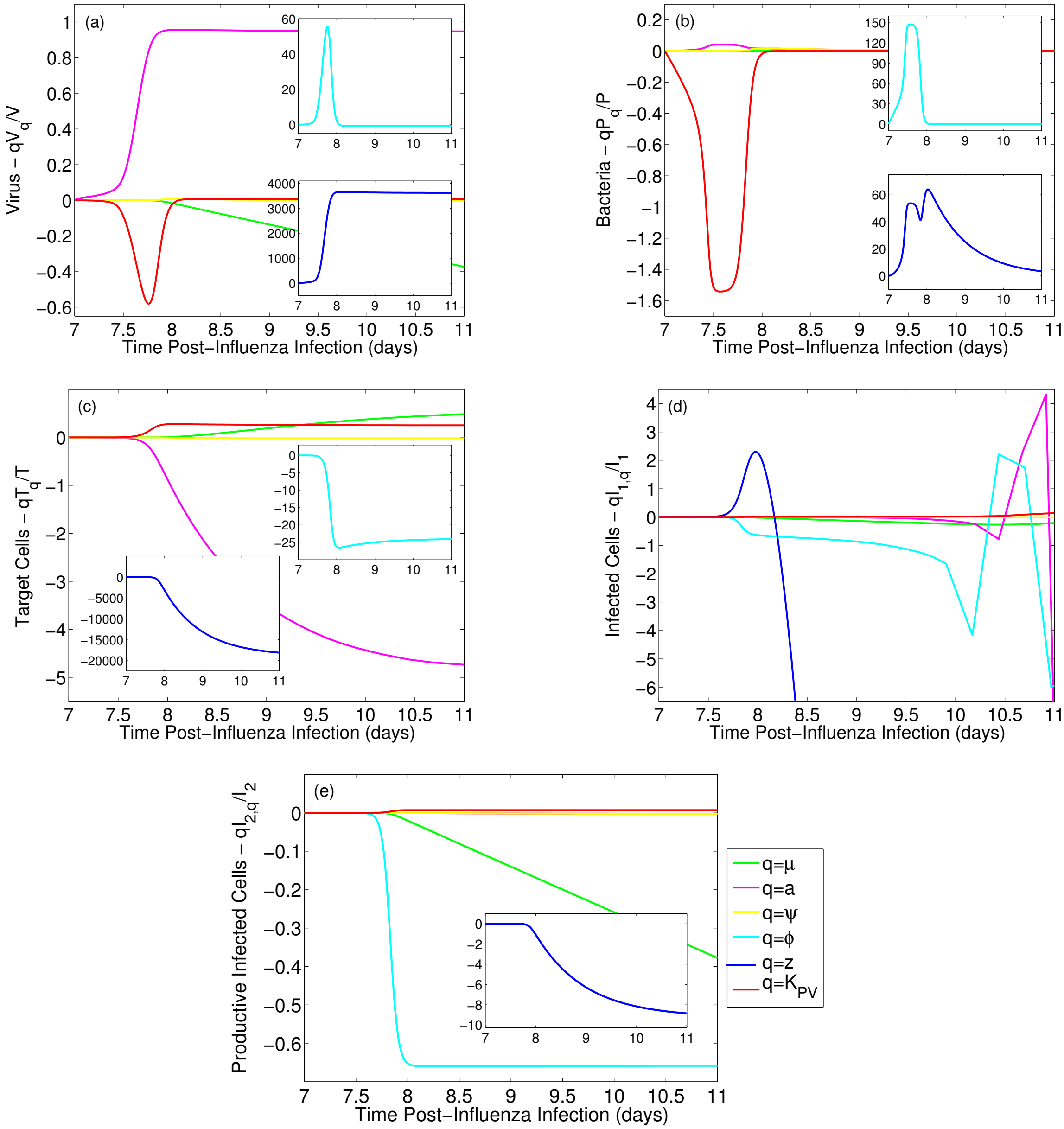

Figure S17: Logarithmic sensitivity solutions for infection with PR8 and 1000 CFU D39. Logarithmic sensitivity solutions for virus (panel (a)), bacteria (panel (b)), target cells (panel (c)), infected cells (panel (d)), and productive infected cells (panel (e)) for the 6 coinfection parameters (μ, z, a, ψ, ϕ, K_{PV}) with $P_0 = 10^2$ CFU/ml and PR8 preinfection.

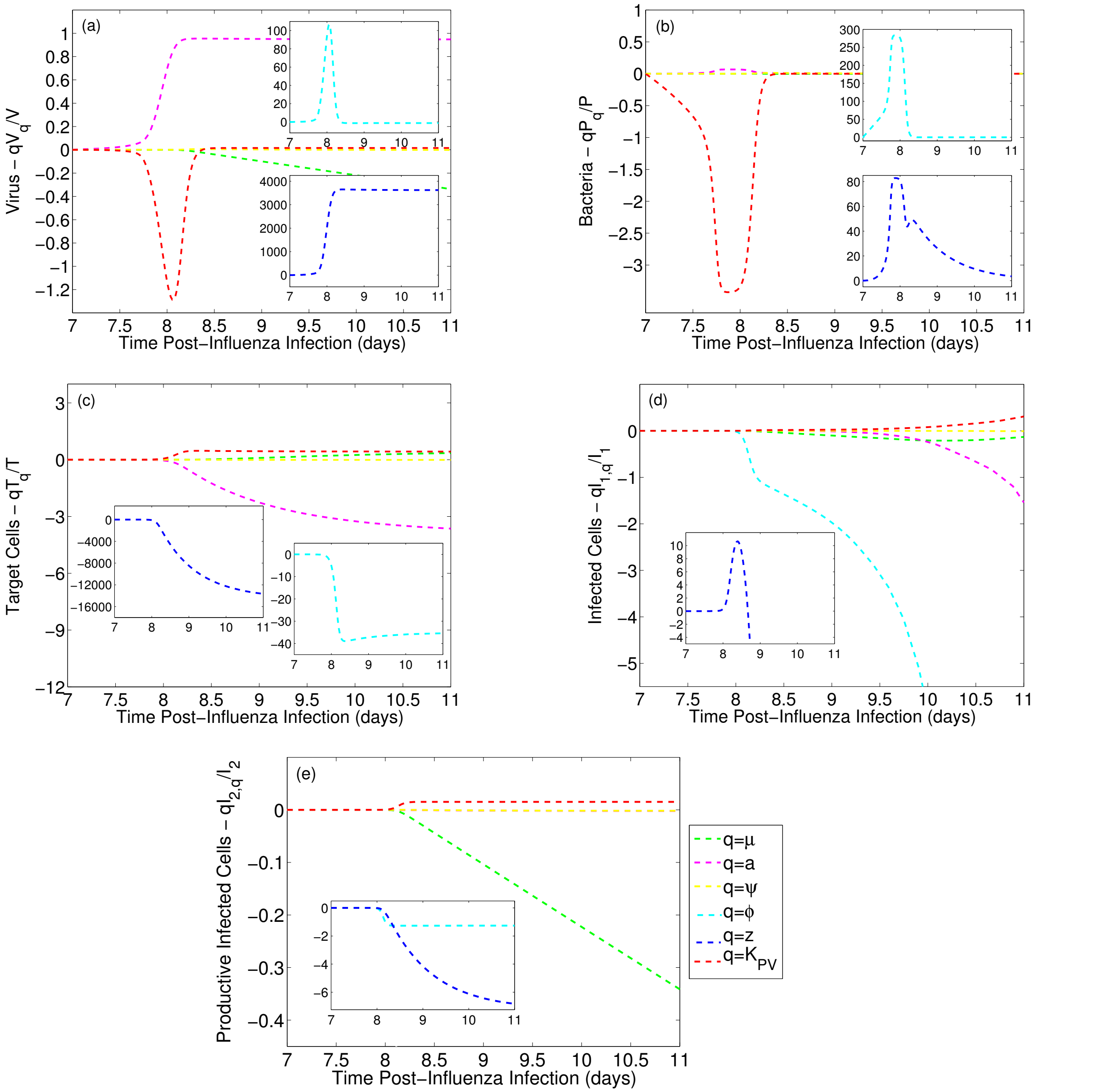

Figure S18: Logarithmic sensitivity solutions for infection with PR8 and 100 CFU D39. Logarithmic sensitivity solutions for virus (panel (a)), bacteria (panel (b)), target cells (panel (c)), infected cells (panel (d)), and productive infected cells (panel (e)) for the 6 coinfection parameters (μ, z, a, ψ, ϕ, K_{PV}) with $P_0 = 10^1$ CFU/ml and PR8 preinfection.

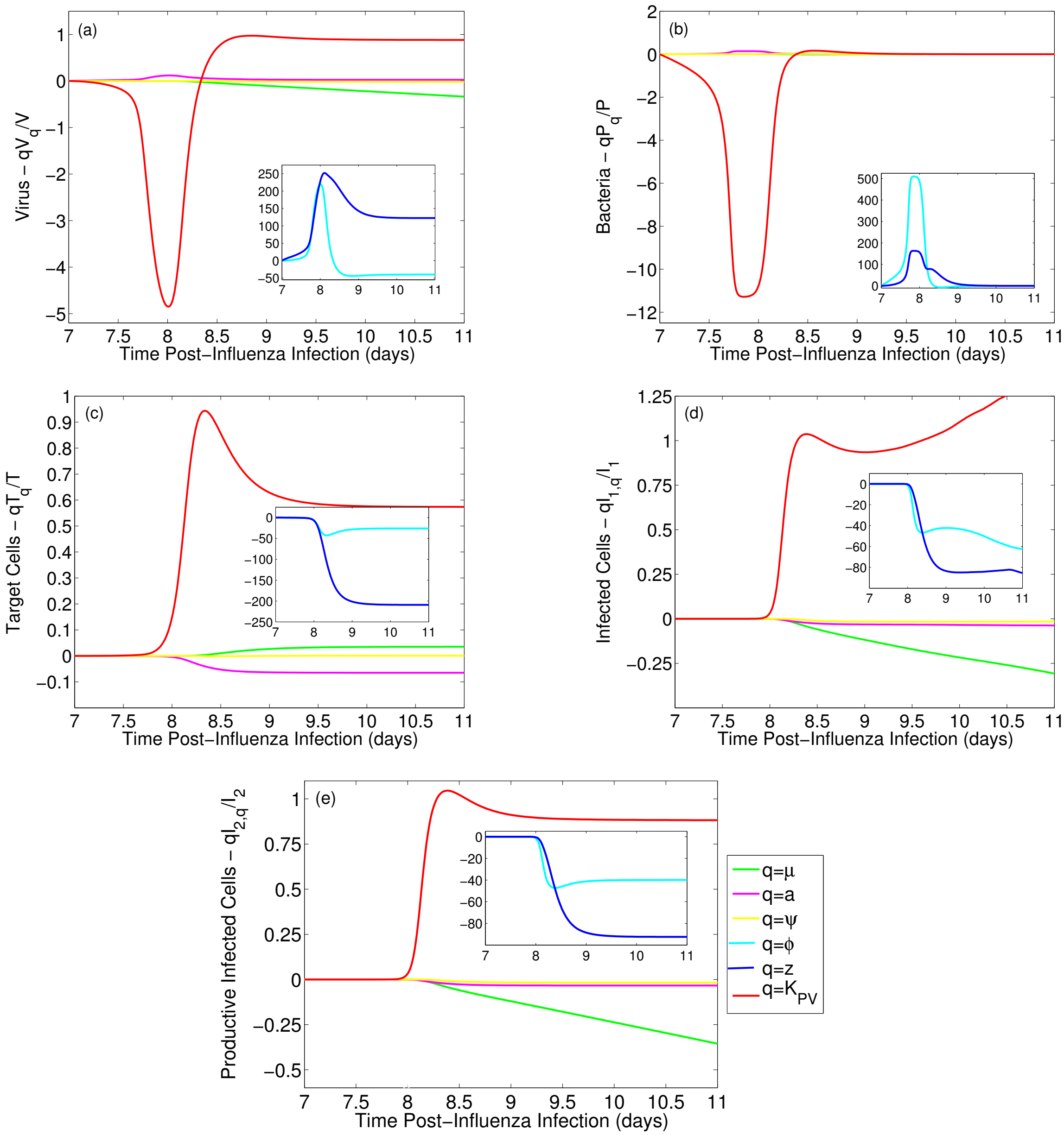

Figure S19: Logarithmic sensitivity solutions for infection with PR8-PB1-F2(1918) and 1000 CFU D39. Logarithmic sensitivity solutions for virus (panel (a)), bacteria (panel (b)), target cells (panel (c)), infected cells (panel (d)), and productive infected cells (panel (e)) for the 6 coinfection parameters (μ, z, a, ψ, ϕ, K_{PV}) with $P_0 = 10^2$ CFU/ml and PR8-PB1-F2(1918) preinfection.
